# Van Vleck analysis of angularly distorted octahedra using *VanVleckCalculator*

**DOI:** 10.1107/S1600576723009925

**Published:** 2024-02-01

**Authors:** Liam. A. V. Nagle-Cocco, Siân E. Dutton

**Affiliations:** a Cavendish Laboratory, University of Cambridge, J. J. Thomson Avenue, Cambridge CB3 0HE, United Kingdom; The University of Western Australia, Australia

**Keywords:** crystal structure, Jahn–Teller distortion, octahedral distortion, octahedral tilting, crystallographic software

## Abstract

Van Vleck modes describe all possible displacements of octahedrally coordinated ligands about a core atom. They are a useful analytical tool for analysing the distortion of octahedra, particularly for first-order Jahn–Teller distortions, but determination of the Van Vleck modes of an octahedron is complicated by the presence of angular distortion of the octahedron. This problem is most commonly resolved by calculating the bond distortion modes (*Q*_2_, *Q*_3_) along the bond axes of the octahedron, disregarding the angular distortion and losing information on the octahedral shear modes (*Q*_4_, *Q*_5_ and *Q*_6_) in the process. In this paper, the validity of assuming bond lengths to be orthogonal in order to calculate the Van Vleck modes is discussed, and a method is described for calculating Van Vleck modes without disregarding the angular distortion. A Python package for doing this, *VanVleckCalculator*, is introduced and some examples of its use are given. Finally, it is shown that octahedral shear and angular distortion are often, but not always, correlated, and a parameter η is proposed as the shear fraction. It is demonstrated that η can be used to predict whether the values will be correlated when varying a tuning parameter such as temperature or pressure.

## Abstract 2

A method and associated Python script, *VanVleckCalculator*, are described for parameterizing octahedral shear and first-order Jahn–Teller distortions in crystal structures.

## 1. Introduction

The Van Vleck distortion modes (Van Vleck, 1939 ▸) describe all possible displacements of octahedrally coordinated ligands about a core atom. They are particularly useful in the context of the Jahn–Teller (JT) effect (Jahn & Teller, 1937 ▸), which in general occurs when a high-symmetry coordination is de­stabilized with respect to a deviation to lower symmetry as a consequence of electronic degeneracy. The JT effect distorts the crystal structure via the JT distortion. While the JT distortion is not unique to octahedra in bulk crystalline materials, it was in octahedra that it was first observed experimentally (Bleaney & Bowers, 1952 ▸), and it was in materials with JT-distorted octahedra that colossal magneto­resistance (Millis *et al.*, 1996 ▸) and high-temperature superconductivity (Fil *et al.*, 1992 ▸; Keller *et al.*, 2008 ▸) were discovered.

**Figure 1:**
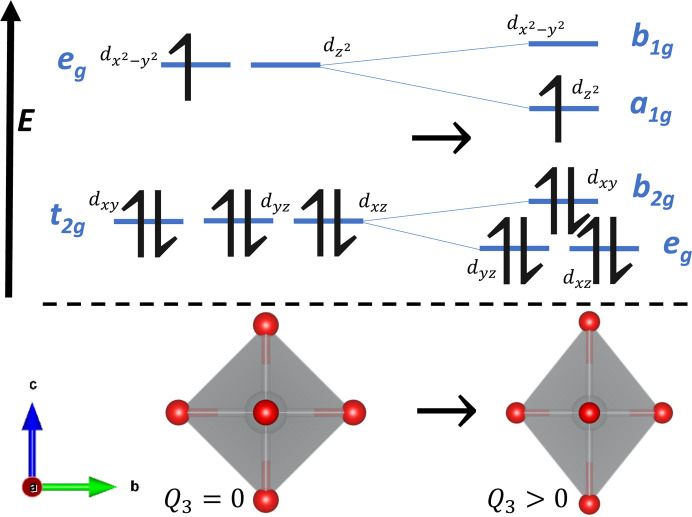
The orbital rearrangement due to a tetragonal elongation for an octahedrally coordinated low-spin *d* ^7^ transition metal ion, which typically occurs as a result of the first-order JT effect.

A transition metal cation in an octahedral configuration will have its *d* orbitals split into three *t*_2*g*_ orbitals at lower energy and two *e*_*g*_ orbitals at higher energy. [Here we use the notation that lower-case symmetry descriptors (such as *e*_*g*_ or *t*_2*g*_) refer to orbitals with this symmetry and upper-case descriptors (such as *E*_*g*_ or *T*_2*g*_) refer to the symmetry more generally.] It will have a number *n* of electrons in these *d* orbitals (hereafter described as *d* ^*n*^). For certain values of *n* and, where applicable, certain low- or high-spin characters,^1^ there will exist multiple orbitals that could be occupied by an electron or an electron hole with equal energy. This degeneracy is destabilizing, resulting in the most stable configuration of atomic sites being one in which the ligands distort from their high-symmetry positions in order to rearrange the orbitals into a non-degenerate system with minimized energy. This is shown for a low-spin *d* ^7^ transition metal cation (such as Ni^3+^ or Co^2+^) in Fig. 1 ▸, though such distortions may occur for any value of *n* in *d* ^*n*^ where there is a degenerate occupancy. The stabilization energy due to the JT effect is larger for *e*_*g*_ degeneracy than *t*_2*g*_ degeneracy, and so the effect will remain prominent up to higher temperatures, and is hence more widely studied, in JT-active materials with *e*_*g*_ degeneracy (Castillo-Martínez *et al.*, 2011 ▸).

In the literature, various techniques for parameterizing the JT distortion are used. An often-used example (Kimber, 2012 ▸; Lawler *et al.*, 2021 ▸; Nagle-Cocco *et al.*, 2022 ▸; Genreith-Schriever *et al.*, 2023 ▸) is the bond-length distortion index, defined by Baur (1974 ▸) as 

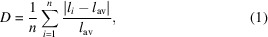

where *n* is the number of ligands (6 for an octahedron), *l*_*i*_ is the distance between the core ion and the *i*th coordinated ion, and *l*_av_ is the average of all the distances between the core ion and coordinated ions.

A similar parameter (Shirako *et al.*, 2012 ▸; Sarkar *et al.*, 2018 ▸; Nagle-Cocco *et al.*, 2022 ▸) is the effective coordination number ECoN, which for an octahedron deviates from 6 only when there is bond-length distortion. ECoN is defined by Hoppe (1979 ▸) as

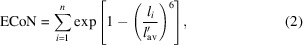

where 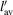 is a modified average distance defined as

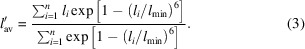

Here, *l*_min_ is the smallest core–ligand bond length in the octahedron.

Finally, a third parameter used to quantify the JT distortion (Schofield *et al.*, 1997 ▸; Kyono *et al.*, 2015 ▸; Mikheykin *et al.*, 2015 ▸) is the quadratic elongation 〈λ〉, defined by Robinson *et al.* (1971 ▸) as

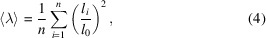

where *l*_0_ is the centre-to-vertex distance of a regular polyhedron of the same volume.

More recently, an alternative approach to modelling polyhedral distortion has been described (Cumby & Attfield, 2017 ▸), involving fitting an ellipsoid to the positions of the ligands around a coordination polyhedron, calculating the three principal axes of the ellipsoid *R*_1_, *R*_2_ and *R*_3_, where *R*_1_ ≤ *R*_2_ ≤ *R*_3_, and using the variance of these three radii as a metric for the distortion. This has been applied to the first-order JT distortion by Pughe *et al.* (2023 ▸).

These parameterizations each have merits. However, they are not sensitive to the symmetry of the octahedral distortion. The Van Vleck modes are conceptually different from each of these for quantifying the JT distortion because they can be used to quantify distortion with the precise symmetry of the transition metal *e*_*g*_ orbitals. This is important because JT distortions typically follow a particular symmetry. When the distortion is due to degeneracy in the *e*_*g*_ orbitals it will be of *E*_*g*_ symmetry; when it is due to degeneracy in the *t*_2*g*_-degenerate orbitals it may be either *E*_*g*_ or *T*_2*g*_ symmetry (Child & Roach, 1965 ▸; Bacci *et al.*, 1975 ▸; Holland *et al.*, 2002 ▸; Halcrow, 2009 ▸; Teyssier *et al.*, 2016 ▸; Schmitt *et al.*, 2020 ▸; Streltsov *et al.*, 2022 ▸), although there is relatively little unambiguous experimental evidence for a JT-induced shear compared with more typical *E*_*g*_ distortion.

In this paper, we present a Python (Van Rossum & Drake, 2009 ▸) package, *VanVleckCalculator*, for calculating the Van Vleck distortion modes. We show that the approach to calculating the modes which is commonly used in the literature is a reasonable approximation for octahedra with negligible angular distortion, but results in the loss of information in other cases. We propose a new metric, the shear fraction η, for understanding the correlation between octahedral shear and angular distortion. Finally, we re-analyse some previously published data in terms of the Van Vleck modes to show that these can be an effective way of understanding octahedral behaviour.

## 2. Theory

Within an octahedron, we can split the six ligand ions into three pairs, where the two ions within the pair are opposite one another. In the absence of angular distortion (*i.e.* assuming all ligand–core–ligand angles are an integer multiple of 90°), there would exist a basis where each of the three axes exist directly along the *x*, *y* and *z* axes, and where the origin in space is defined as the centre of the octahedron.

**Figure 2:**
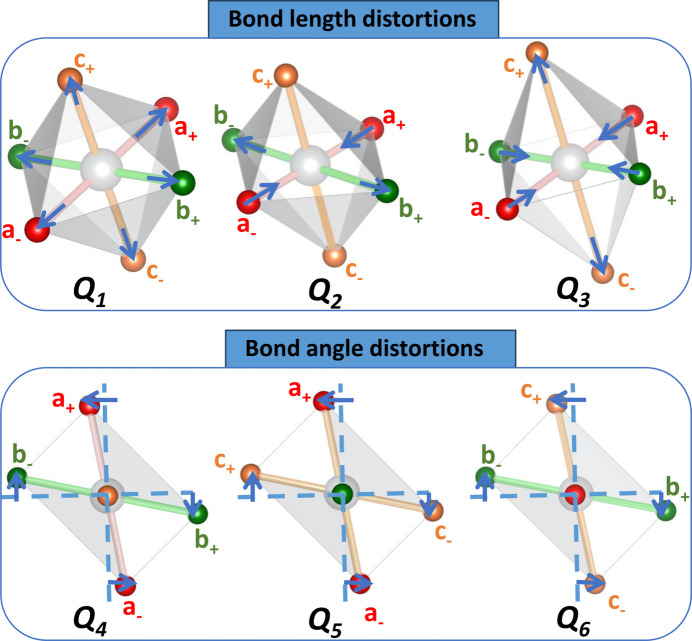
The six Van Vleck modes exhibited for an octahedron, with sites labelled using the notation in the *Theory* [Sec sec2] section. For the octahedra exhibiting *Q*_1_, *Q*_2_ and *Q*_3_ distortions, there is no angular distortion; for the octahedra exhibiting *Q*_4_, *Q*_5_ and *Q*_6_ distortions, there is no bond-length distortion. For the octahedral shear (*Q*_4_, *Q*_5_ and *Q*_6_) modes, axes are drawn to show where the bond directions would be if undistorted. An octahedron can exhibit several, or all, of these distortions simultaneously.

Each pair within an octahedron can therefore be assigned to an axis and labelled as the *a*, *b* or *c* pair, respectively. Within a pair, ions can be labelled as − or + depending on whether they occur at a negative or positive displacement from the origin, along the axis, respectively. This notation is demonstrated in Fig. 2 ▸, where each pair of ions is represented by a different colour.

For each of the six ligands, we define a set of coordinates 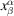 , 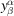 and 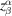 , where α is *a*, *b* or *c* denoting the pair in which the ligand is, and β is − or + denoting the ion within the pair.

The ideal positions of the six ligands are (*R*, 0, 0), (−*R*, 0, 0), (0, *R*, 0), (0, −*R*, 0), (0, 0, *R*) and (0, 0, −*R*), where *R* is defined as the distance between the centre of the octahedron and the ligand in an ideal octahedron (in practice, this is taken as the average of the core–ligand bond distances). This results in 18 independent variables. Using these, we further define a set of Van Vleck coordinates (capitalized to distinguish from true coordinates) which give the displacement of the ion within an axis away from its ideal position. For instance, for the ion with α = *a* and β = −, 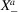 = 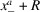 , 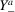 = 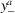 and 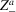 = 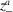 . See Fig. 2 ▸ for clarification of the ion notation.

Using these coordinates, the first six Van Vleck modes (*Q*_*j*_, *j* = 1–6) are defined as follows (Van Vleck, 1939 ▸): 

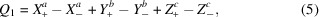




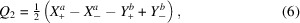




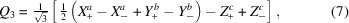




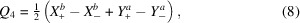




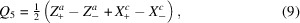




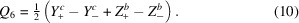

We only discuss these first six Van Vleck modes, which are shown in Fig. 2 ▸. *Q*_1_ to *Q*_3_ describe bond-length distortions, whereas *Q*_4_ to *Q*_6_ describe octahedral shear distortions. *Q*_1_ is a simple expansion/contraction mode which does not affect symmetry and will not be discussed further.

**Figure 3:**
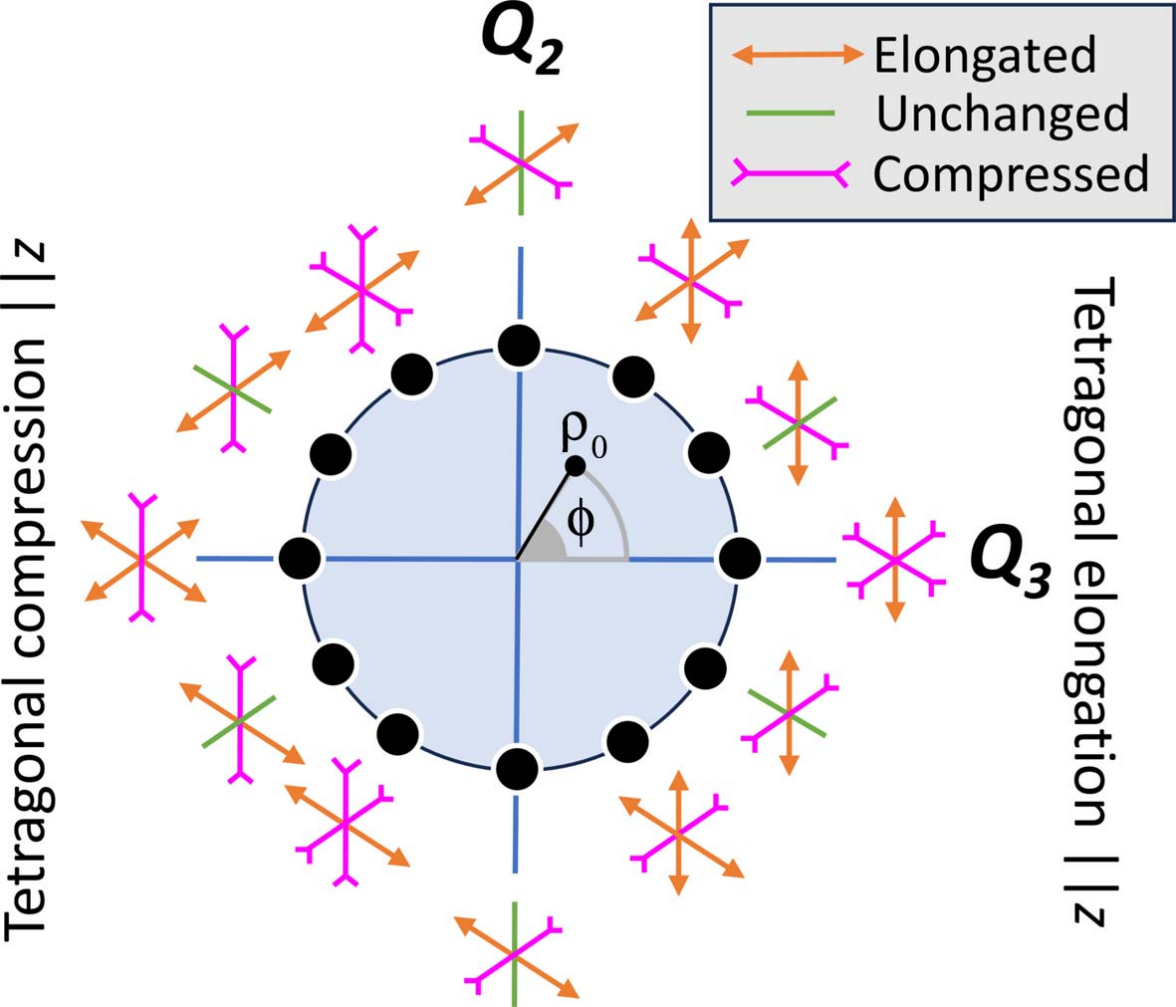
The *Q*_2_–*Q*_3_ phase space for elongated octahedra, with a representation of the values ρ_0_ and ϕ. Based on a figure from an article by Goodwin (2017 ▸).

*Q*_2_ and *Q*_3_ are a planar rhombic distortion and a tetragonal distortion, respectively; they are considered degenerate due to the Hamiltonian, which is discussed for instance by Kanamori (1960 ▸). These two modes form a basis for distortions describing different octahedral configurations with the symmetry of the transition metal *e*_*g*_ orbitals (Goodenough, 1998 ▸; Khomskii & Streltsov, 2021 ▸). These modes are of most relevance for first-order JT distortions occurring due to degenerate *e*_*g*_ orbitals. A phase space of possible octahedral configurations can be constructed using these two parameters (Kanamori, 1960 ▸), as shown in Fig. 3 ▸. Here the magnitude of the distortion ρ_0_ can be calculated as 

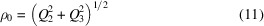

and the angle ϕ of this distortion, being of purely *Q*_3_ character, can be calculated by

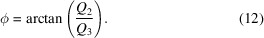

(Note that this angle does not represent a physical angle within the octahedron.)

All possible combinations of the *Q*_2_ and *Q*_3_ modes correspond to a particular angle ϕ and hence a particular configuration as shown in Fig. 3 ▸. The structural effect of a rotation of ϕ within a range of 120° can be quite significant, as shown in Fig. 3 ▸; such changes can manifest as a JT-elongated (compressed) octahedron with four short (long) and two long (short) bonds [such as NiO_6_ in NaNiO_2_ (Nagle-Cocco *et al.*, 2022 ▸)] or two short, two medium and two long bonds [such as LaMnO_3_ (Rodriguez-Carvajal *et al.*, 1998 ▸)].

A characteristic of JT distortion is that, in the absence of external distortive forces, the symmetry of the structure matches the symmetry of the orbitals involved. Typically, any *d*-orbital JT distortion will have some planar rhombic (*Q*_2_) or tetragonal (*Q*_3_) character. However, sometimes when the degeneracy occurs in the *t*_2*g*_ orbital, there may instead be a trigonal component to the symmetry of the distortion, which manifests as an angular distortion instead (Child & Roach, 1965 ▸; Bacci *et al.*, 1975 ▸; Holland *et al.*, 2002 ▸; Halcrow, 2009 ▸; Teyssier *et al.*, 2016 ▸; Schmitt *et al.*, 2020 ▸; Streltsov *et al.*, 2022 ▸). For the more commonly studied case of a degeneracy in the *e*_*g*_ orbitals, the effect of a rotation of ϕ similarly changes the symmetry of the *d* orbitals. Fig. 1 ▸ shows the splitting of the *d* orbitals in an octahedrally coordinated *d* ^7^ transition metal due to an elongation-type first-order JT distortion, where the tetragonal elongation occurs along the *z* axis. Note that the unpaired *e*_*g*_ electron occupies the 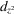 orbital. In the opposite case of a compression-type first-order JT distortion along the *z* axis, the lower-energy, and hence singly occupied, orbital would be the 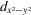 ; this would correspond to a rotation in ϕ of 180°. More generally, as a function of ϕ, there exist a set of special angles separated by a 60° rotation corresponding to a particular *e*_*g*_ orbital being singly occupied by a *d* electron. These are tabulated in Table 1 ▸. An octahedron for which ϕ does not correspond to one of these special angles exhibits orbital mixing (Rodriguez-Carvajal *et al.*, 1998 ▸; Zhou & Goodenough, 2008*b* ▸).

The *Q*_4_ to *Q*_6_ modes describe shear of the octahedra, *i.e.* the effect whereby paired ligands on opposite sides of a central ion are displaced in opposite directions and have trigonal *T*_2*g*_ character. The shear modes may be used to quantify the JT distortion in octahedra where the degeneracy occurs within *t*_2*g*_ orbitals (Child & Roach, 1965 ▸; Teyssier *et al.*, 2016 ▸). The magnitude of the calculated shear is typically correlated with angular distortion, which is commonly quantified using the 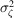 metric called the bond-angle variance (BAV) (Robinson *et al.*, 1971 ▸), defined here as 

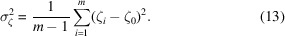


*m* is the number of bond angles (*i.e.* 12 for an octa­hedron), ζ_*i*_ is the *i*th bond angle and ζ_0_ is the ideal bond angle for a regular polyhedron (*i.e.* 90° for an octahedron). However, for direct comparison with the shear modes, it is more appropriate to use the standard deviation σ_ζ_.

**Figure 4:**
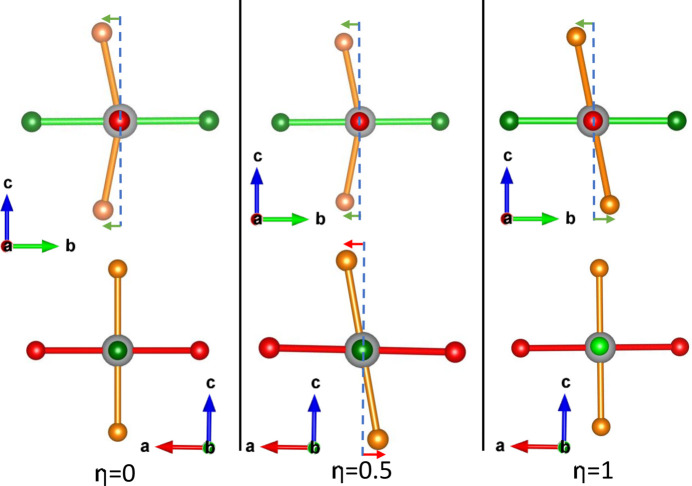
Three possible octahedral shear/anti-shear distortions, with the associated value of the shear fraction η as defined in equation (22). In the case where η = 0, the only distortion is anti-shear within a single plane. In the case where η = 0.5, there are two planes in which there is distortion, a shear and an anti-shear distortion equal in magnitude. In the case where η = 1, there is a plane with a purely shear distortion.

For an octahedron with non-zero *T*_2*g*_(*Q*_4_, *Q*_5_, *Q*_6_) modes, increasing their magnitude will increase the angular distortion, but an octahedron may have angular distortion without exhibiting octahedral shear. To analyse the extent to which angular distortion in an octahedron is due to shear, we propose a shear fraction parameter η, demonstrated in Fig. 4 ▸ and defined below.

First, we must define a set of shear and ‘anti-shear’ angular indices, which are modifications of equations (8) to (10 [Disp-formula fd10]) in terms of angles rather than displacements. The indices are represented with Δ and a subscript corresponding to the plane in which rotation occurs: the *ab* plane corresponds to the *Q*_4_ mode, the *ac* plane to the *Q*_5_ mode and the *bc* plane to the *Q*_6_ mode. The absence or presence of a prime symbol ′ designates whether the index represents shear or anti-shear, respectively. Finally, the δ angle is the rotation of the ligand from its ideal Van Vleck coordinate in a clockwise direction, within the plane in which the corresponding Van Vleck shear (*Q*_4_ to *Q*_6_) would occur. These are defined thus (see the supporting information, Fig. S7): 

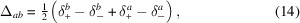




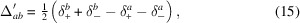




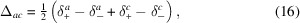




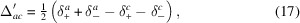




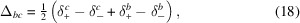




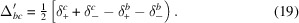




We then quantify the shear and anti-shear distortions using the following equations: 

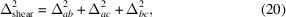




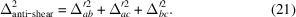




From here, we define the shear fraction η as 

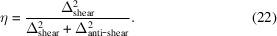

This η parameter will be important in interpreting the relation between the angular distortion σ_ζ_ and the Van Vleck shear modes *Q*_4_ to *Q*_6_.

## 3. Implementation

In this section, the algorithm used to calculate the Van Vleck distortion modes is discussed. It is written using Python 3 (Van Rossum & Drake, 2009 ▸) as a package called *VanVleckCalculator*, with the full code available on GitHub (Nagle-Cocco, 2023 ▸) and also presented with annotations in the supporting information. Data handling and some calculations make use of *NumPy* (Harris *et al.*, 2020 ▸) and crystal structures are handled using *PyMatGen* (Ong *et al.*, 2013 ▸).

A flow chart showing the octahedral rotation algorithm can be found in Fig. S1.

Besides calculating the Van Vleck modes and the angular shear modes described in this paper, *VanVleckCalculator* can also calculate various other parameters as described in the supporting information.

### 3.1. Selecting an origin

Selection of the origin is a key step in calculating Van Vleck modes. The most common approach, for an *MX*_6_ octahedron, is to take the *M* ion as the origin. This is a reasonable approach, given that *M* ions are typically positioned at, or very close to, the centre of an octahedron. This is particularly appropriate for unit cells derived from Rietveld refinement (Rietveld, 1969 ▸) of Bragg diffraction data, where the *M* ion is likely to occur on a high-symmetry Wyckoff site. A third, similar, option would be to choose the average position of the six ligands as the origin in space. An example of when this may be a desirable choice would be for systems exhibiting a pseudo-JT effect (also called the second-order JT effect), where the central cation is offset from the centre of the octahedron.

In some instances, a crystal structure may be simulated using a supercell. Examples include ‘big-box’ pair distribution function (PDF) analysis (Tucker *et al.*, 2007 ▸) and molecular dynamics (MD) (Bocharov *et al.*, 2020 ▸) simulations. Such a supercell typically retains the periodicity which is an axiom of a typical crystallographic unit cell but will exhibit local variations. For instance, a unit cell obtained by analysis of Bragg diffraction data is typically regarded as an ‘average’ structure, insensitive to local phenomena such as thermally driven atomic motion or disordered atomic displacements such as a non-cooperative JT distortion. In a crystallographic unit cell, thermal motion of atoms is typically represented by variable atomic displacement parameters (ADPs) (Peterse & Palm, 1966 ▸). In contrast, a supercell should reflect local phenomena, for instance exhibiting local JT distortions in a system with a non-cooperative JT distortion, and representing thermal effects not with ADPs but rather by distributing equivalent atoms in adjacent repeating units in slightly different positions. In this regard, a supercell can be considered a ‘snapshot’ of a crystal system at a point in time. It may therefore not be appropriate to set the core ion as the centre of the octahedron in a supercell, as the positioning of both core and ligand ions is in part due to thermal effects and so the ‘centre’ of the octahedron will be displaced as a result of random motion. The alternative option would simply be to use the crystallographic site of the central ion and fix this as independent of the precise motion of the central ion locally.

In *VanVleckCalculator*, the user has the option to take as the centre of the octahedron either the central ion, the average position of the six ligands or a specified set of coordinates.

### 3.2. Calculating Van Vleck modes along bond directions

The calculation of the Van Vleck modes, as described in the *Theory* [Sec sec2] section, requires that the basis in space be the octahedral axes (*i.e.* the three orthogonal axes entering the octahedron via one vertex, passing through the central ion and exiting via the opposite vertex). For a given crystal structure, this may require that an octahedron be rotated about each of the three axes making up the basis until the octahedral axes perfectly align with the basis. This becomes more complicated when the octahedron exhibits angular distortion (*i.e.* exhibits ligand–core–ligand angles that are not integer multiples of 90°). In this case, it is impossible to define octahedral axes according to the strict criteria previously defined.

In the literature, this problem is generally evaded by simply calculating the Van Vleck modes on the basis of bond directions rather than Cartesian coordinates; see, for example, previous work on the perovskite LaMnO_3_ (Goodenough *et al.*, 1961 ▸; Rodriguez-Carvajal *et al.*, 1998 ▸; Capone *et al.*, 2000 ▸; Chatterji *et al.*, 2003 ▸; Zhou & Goodenough, 2008*b* ▸; Zhou *et al.*, 2011 ▸; Snamina & Oleś, 2016 ▸; Fedorova *et al.*, 2018 ▸; Lindner *et al.*, 2022 ▸), other perovskites (Alonso *et al.*, 2000 ▸; Wang *et al.*, 2002*a* ▸; Tachibana *et al.*, 2007 ▸; Zhou & Goodenough, 2008*a* ▸; Castillo-Martínez *et al.*, 2011 ▸; Franchini *et al.*, 2011 ▸; Chiang *et al.*, 2011 ▸; Dong *et al.*, 2012 ▸; Fedorova *et al.*, 2015 ▸; Ji *et al.*, 2019 ▸; Xu *et al.*, 2020 ▸; Ren *et al.*, 2021 ▸) or non-perovskite materials (Moron *et al.*, 1993 ▸; Cussen *et al.*, 2001 ▸; Wang *et al.*, 2002*b* ▸) (we note that some reports use a different variation which still uses Kanamori’s approximation; papers cited here include those which use the approximation, even if the precise definitions differ). In this case, *Q*_2_ and *Q*_3_ are defined according to the following equations which were first expressed by Kanamori (1960 ▸), where *l*, *m* and *s* are the short, medium and long bond lengths, respectively [the equations presented here differ from Kanamori’s as they have been multiplied by a factor of 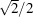 so that they are mathematically equivalent to equations (6) and (7)]: 

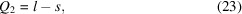




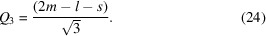




**Figure 5:**
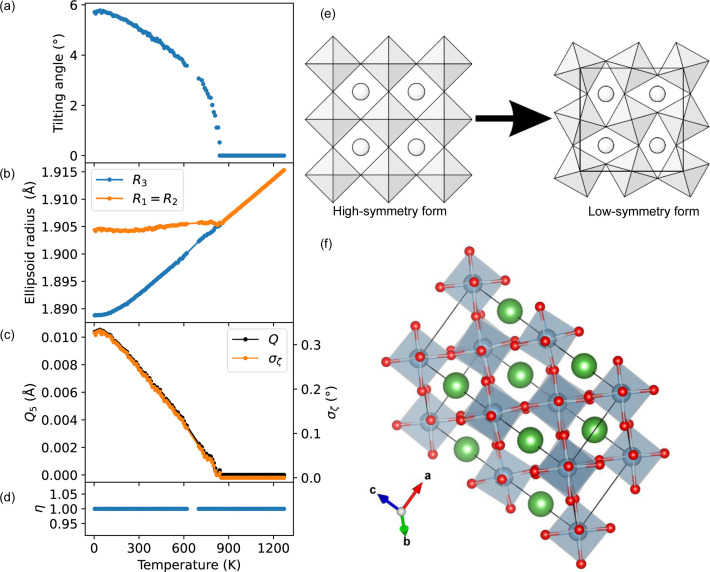
The results of our analysis of LaAlO_3_ as a function of temperature. (*a*) The octahedral tilting angle as reported by Hayward *et al.* (2005 ▸) and extracted using *DataThief III* (Tummers, 2006 ▸). (*b*) The radii of the minimum bounding ellipsoid fitted to the O anions of the AlO_6_ octahedra using *PIEFACE* (Cumby & Attfield, 2017 ▸). (*c*) The octahedral shear parameter *Q*_5_ of the AlO_6_ octahedra, where *Q*_5_ = −*Q*_4_ = −*Q*_6_, calculated using *VanVleckCalculator*, compared with the bond-angle standard deviation σ_ζ_ (orange). (*d*) The shear fraction η, defined in equation (22). (*e*) The transition between low-symmetry (tilting) and high-symmetry (tilt-free) perovskite structures, adapted with permission from Angel *et al.* (2005 ▸), copyright (2005) American Physical Society. (*f*) The perovskite crystal structure of LaAlO_3_ at 4.2 K, drawn using the structure reported by Hayward *et al.* (2005 ▸).

This relies on the implicit assumption that the bond lengths are orthogonal. This is clearly a reasonable approximation in many cases, particularly when the angular distortion is very small. For instance, in LaMnO_3_, the corner-sharing octahedral connectivity enables mismatched polyhedra to tessellate via octahedral tilting [Fig. 5(*e*) in Section 4.1] rather than intra-octahedral angular distortion. However, for systems with greater angular distortion, for instance those with edge- or face-sharing interactions, it is not so clear that this approximation is valid.

### 3.3. Calculating Van Vleck modes within Cartesian coordinates

In *VanVleckCalculator* we have written an algorithm for rotating an octahedron about three Cartesian axes with a defined origin within the octahedron, such that the ligands are as close as possible to the axes (within the constraint that there is angular distortion). This allows for calculation of Van Vleck modes in a way that does not artificially constrain the octahedral shear modes (*Q*_4_, *Q*_5_ and *Q*_6_) to be zero.

First, three orthogonal axes are taken as the *x*, *y* and *z* axes.^2^ By default, these are the [1, 0, 0], [0, 1, 0] and [0, 0, 1] axes, respectively, but alternative sets of orthogonal vectors can be given by the user. For instance, for regular octahedra rotated 45° about the *x* axis, the user would be recommended to give as axes [1, 0, 0], 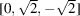 and 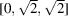 . This vector is given as a Python list with shape (3, 3). For consistency, the cross product of the first two axes should always be parallel with the third given vector; if anti-parallel, the algorithm will automatically multiply all elements in the third vector by −1. The three pairs of the octahedron (as defined in the *Theory* [Sec sec2] section) are each assigned to one of these three axes. This assignment is performed according to the magnitude of the projection of a vector between the two atoms in a pair along a given axis, with the *z* axis assigned first, then the *y* axis from amongst the two pairs not assigned to the *z* axis, and finally the *x* axis is automatically assigned to the remaining pair. Within each pair, the ligands are then ordered such that the ligand with the negative distance is the first along the assigned vector and the ligand with the positive distance occurs second.

Second, the octahedron is rotated repeatedly about the *x*, *y* and *z* directions of the basis until the orthogonal axes supplied in the previous step match the basis precisely. This is performed in a ‘while’ loop structure, with the rotation angles about the three axes summed in quadrature and compared with a defined tolerance (by default, 3 × 10^−4^ rad in *Van­VleckCalculator*); if the total rotation exceeds the tolerance, the step is repeated.^3^ This step is usually unnecessary and can be skipped by leaving the default set of orthogonal axes, which are [1, 0, 0], [0, 1, 0] and [0, 0, 1] (meaning no rotation will occur).

Third, an automatic rotation algorithm will further minimize the effect of angular distortion. For each of the three axes, the four ligands not intended to align with that axis are selected. The angle to rotate these four ligands about the origin such that each is aligned with its intended axis within the plane perpendicular to the axis of rotation is calculated. The octahedron is then rotated about this axis by the average of these four angles. This occurs iteratively until, for a given iteration, the sum (in quadrature) of the three rotation angles is less than the already-mentioned defined tolerance.

At this point, the octahedron is optimally aligned with the basis (given the limitation that there may be angular distortion) and the Van Vleck modes can be calculated.

### 3.4. Ignoring or including angular distortion: a comparison

To evaluate the utility of calculating the Van Vleck modes without disregarding the angular distortion, we perform a comparison between the two approaches. We have calculated the Van Vleck distortion modes and associated parameters for octahedra in NaNiO_2_ and LaMnO_3_ with both a method that ignores angular distortion and calculates modes along bond directions [consistent with the *Q*_2_ and *Q*_3_ equations defined by Kanamori (1960 ▸)] and a method that uses Cartesian coordinates in order to take angular distortion into account. Table 2 ▸ shows this for these two materials. Firstly, for the Van Vleck modes calculated without ignoring angular distortion, we can see that the octahedral shear modes (*Q*_4_, *Q*_5_, *Q*_6_) are larger for the material with higher angular distortion (as quantified using bond-angle variance). While the effect of ignoring angular distortion is significant for the *Q*_4_, *Q*_5_ and *Q*_6_ modes, it makes negligible difference for the calculation of *Q*_2_ and *Q*_3_ modes and the associated ρ_0_ and ϕ parameters. It is therefore likely to be a reasonable approximation to take, particularly for calculation of ϕ as is common in the literature, even for octahedra which exhibit higher angular distortion. However, there is a definite loss of information in assuming that the shear modes *Q*_4_ to *Q*_6_ are zero. The impact of this is assessed in the case studies.

## 4. Case studies

### 4.1. Temperature dependence of octahedral shear in LaAlO_3_

Perovskite and perovskite-like crystal structures are amongst the most important and most widely studied crystalline material classes in materials science today. Perovskite crystal structures have *ABX*_3_ chemical formulae, with *A* and *B* being ions at the centres of dodecagons and octahedra, respectively, and the *X* anions constituting the vertices of these polyhedra. The *BX*_6_ octahedra interact via corner-sharing interactions. There are also perovskite-like crystal structures such as the double perovskites, *A*_2_ *BB*′*X*_6_ (King & Woodward, 2010 ▸; Koskelo *et al.*, 2023 ▸), for which many of the same principles apply.

The ideal perovskite system would be cubic, with space group 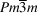 , but many related structures with lower symmetry are known. This typically occurs in three situations (Woodward, 1997 ▸):

(i) when there is a mismatch between the ionic radii of the octahedrally coordinated *B* cation and the dodecagonally coordinated *A* cation, resulting in tilting of the octahedra [see Fig. 5 ▸(*e*)];

(ii) when there is displacement of the central cation from the centre of the octahedron, typically due to the pseudo-JT effect;

(iii) when the ligands of the octahedron are distorted by electronic phenomena such as the first-order JT effect.

In this case study, we focus on the first case, where a size mismatch results in octahedral tilting. Octahedra are often modelled as rigid bodies, but in practice they are not rigid in all systems and the octahedral tilting will often induce strain resulting in angular distortion. This is typically far smaller than that seen in edge-sharing materials such as NaNiO_2_, but it is large enough that it cannot be disregarded when attempting a full understanding of the structure of the material. As was noted by Darlington (1996 ▸), this angular distortion commonly manifests as shear.

LaAlO_3_ is a perovskite-like *ABX*_3_ material which is cubic (space group 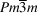 ) above around ∼830 K but exhibits a rhombohedral distortion below this temperature (with space group 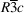 ) due to octahedral tilting (Hayward *et al.*, 2005 ▸) [Figs. 5 ▸(*e*) and 5 ▸(*f*)]. There is no bond-length distortion in either temperature regime; a calculation of the bond-length distortion index would yield a value of zero at all temperatures. In the low-temperature regime, the magnitude of the distortion continuously decreases with increasing temperature, reaching zero at the transition temperature. Most commonly in the literature, the tilting angle between the octahedral axis and the *c* axis (0° in the cubic phase) is used to quantify this distortion; for LaAlO_3_ this is shown in Fig. 5 ▸(*a*). The strain induced by this distortion results in intra-octahedral angular distortion. Hayward *et al.* (2005 ▸) modelled this in terms of strain tensors, finding a linear temperature dependence below the transition temperature. This differs from the temperature dependence of the tilting angle (which resembles an exponential decline), implying the two are related but distinct phenomena. Cumby & Attfield (2017 ▸) instead modelled the octahedral distortion for this same data set using the radii of a minimum-bounding ellipsoid and also found an approximately linear temperature dependence of the long and short radii as they approach convergence [see Fig. 5 ▸(*b*)].

Here, we calculate the Van Vleck shear modes. Due to the symmetry of the octahedral tilting, there is only one independent shear mode and *Q*_5_ = −*Q*_4_ = −*Q*_6_. We compare this with the bond-angle standard deviation given in equation (13) [Fig. 5 ▸(*c*)]. We see that, despite being distinct parameters, they have identical temperature dependences. We attribute this to the shear fraction η being precisely 1 for all temperatures where there is angular distortion, meaning that shear is completely correlated with angular distortion.

### 4.2. Big-box analysis of PDF data on LaMnO_3_

**Figure 6:**
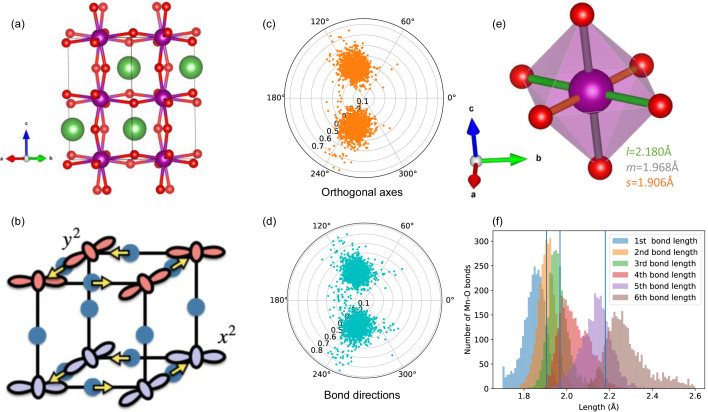
(*a*) The perovskite-like structure of LaMnO_3_ as obtained from ICSD structure 50334. (*b*) The orbital ordering at room temperature in LaMnO_3_. Reprinted with permission from Khomskii & Streltsov (2021 ▸), copyright (2021) American Chemical Society. (*c*) and (*d*) Polar plots with each point representing the calculated ϕ and ρ_0_ values for each MnO_6_ octahedron in a 10 × 10 × 8 supercell of LaMnO_3_ at room temperature, as obtained from reverse Monte Carlo analysis of neutron PDF data collected by Thygesen *et al.* (2017 ▸). In panel (*c*), orthogonal axes were used (*i.e.* angular distortion was included in the calculation, using the method described in this manuscript), whereas in panel (*d*) the Mn—O bond directions were taken as the axes, regardless of orthogonality. (*e*) The Mn^3+^ octahedron which exhibits a mixed *Q*_2_–*Q*_3_-type distortion due to the first-order JT effect, manifesting as three different bond lengths, labelled in ascending order of length as *s* (orange), *m* (grey) and *l* (green). (*f*) A histogram of the smallest to largest Mn—O bond lengths within each octahedron in the 10 × 10 × 8 supercell, with the blue vertical lines indicating the bond lengths in the average structure.

The JT distortion in LaMnO_3_, a perovskite-like *ABX*_3_ material which has the crystal structure shown in Fig. 6 ▸(*a*), occurs as a consequence of degeneracy in the *e*_*g*_ orbitals on the high-spin *d* ^4^ Mn^3+^ ion. At ambient temperatures it is a prime example of a cooperative JT distortion, exhibiting long-range orbital order where the elongation of the JT axis alternates between the *a* and *b* directions for neighbouring MnO_6_ octahedra, never occurring along the *c* direction (Khomskii & Streltsov, 2021 ▸) [Fig. 6 ▸(*b*)]. With heating to ∼750 K, the JT distortion can no longer be observed in the average structure obtained from Bragg diffraction (Rodriguez-Carvajal *et al.*, 1998 ▸). However, the JT distortion persists locally, as has been shown by PDF (Qiu *et al.*, 2005 ▸) and EXAFS (García *et al.*, 2005 ▸; Souza *et al.*, 2005 ▸) measurements. This transition is one of the most widely studied orbital order–disorder transitions for first-order JT distortion. The high-temperature orbital regime has been described theoretically in terms of a three-state Potts model (Ahmed & Gehring, 2006 ▸, 2009 ▸), a view supported by big-box analysis of combined neutron and X-ray PDF data (Thygesen *et al.*, 2017 ▸), as performed using *RMCProfile* (Tucker *et al.*, 2007 ▸).

In this case study, we take a 10 × 10 × 8 supercell of LaMnO_3_, obtained using *RMCProfile* against total scattering data obtained at room temperature and previously published in the aforementioned work (Thygesen *et al.*, 2017 ▸). The results are shown in Fig. 6 ▸. We repeat the analysis of this supercell from the perspective of the *E*_*g*_(*Q*_2_, *Q*_3_) Van Vleck distortion modes, using two different approaches: (i) applying the algorithm for automatically determining a set of orthogonal axes to each octahedron individually, and (ii) following the Van Vleck equations (23) and (24) proposed by Kanamori (1960 ▸) where angular distortion is disregarded. In each of these cases the crystallographic site of the supercell is taken as the origin and so thermally driven variations in the Mn position will not affect the result.

As can be seen in Figs. 6 ▸(*c*) and 6 ▸(*d*), there are two clusters of octahedra within the polar plot, occurring at ϕ ≃ ±107°. This corresponds to occupation of the 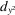 orbitals (+) and of the 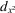 orbitals (−). In both cases, the superposition of perpendicular *Q*_3_ compression and elongation modes results in an octa­hedron with mixed *Q*_2_–*Q*_3_ character. This finding is consistent with previous work which placed MnO_6_ octahedra from LaMnO_3_ onto the framework of an *E*_*g*_(*Q*_2_, *Q*_3_) polar plot (Zhou & Goodenough, 2008*a* ▸; Zhou *et al.*, 2011 ▸).

Fig. 6 ▸(*e*) shows the MnO_6_ octahedron in the average structure of LaMnO_3_ at room temperature, with the three different bond lengths plotted in Fig. 6 ▸(*f*) along with a histogram of all the bond lengths in the supercell. This shows how the combination of the *Q*_2_ and *Q*_3_ distortion modes manifests in the octahedral distortion.

The *Q*_2_ contribution to the distortion, as seen from the three different Mn—O bond lengths in LaMnO_3_, is also present in JT-distorted *A*CuF_3_ (*A* = Na, K, Rb) (Lufaso & Woodward, 2004 ▸; Marshall *et al.*, 2013 ▸; Khomskii & Streltsov, 2021 ▸) and even in some JT-undistorted perovskites (Zhou & Goodenough, 2008*a* ▸), indicating it is related to the structure. It is not intrinsic to JT-distorted manganates, as it is absent in high-spin *d* ^4^ Mn^3+^ with edge-sharing octahedral interactions and col­linear orbital ordering such as α-NaMnO_2_ and LiMnO_2_ [checked using ICSD (Inorganic Crystal Structure Database, FIZ-Karlsruhe, Germany; https://icsd.fiz-karlsruhe.de/index.xhtml) references 15769 (Jansen & Hoppe, 1973 ▸) and 82993 (Armstrong & Bruce, 1996 ▸) respectively]. The *Q*_2_ component to the octahedral distortion is therefore probably intrinsic to the crystal structure (Zhou & Goodenough, 2006 ▸, 2008*a* ▸), which occurs as a result of octahedral tilting reducing the symmetry from cubic 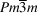 to *Pnma*. In LaMnO_3_, the combination of the *Q*_2_ component to the distortion and the orbital ordering [Fig. 6 ▸(*b*)] are a possible distortion of the *Pnma* space group. In this way, the orbital ordering may be coupled to the octahedral tilting, a link previously made by Lufaso & Woodward (2004 ▸).

Finally, we also calculate the *Q*_4_ to *Q*_6_ octahedral shear modes for all octahedra in the supercell, presented as a histogram in Fig. S2. We present the average and standard deviation, calculated assuming orthogonal axes and with the automated octahedral rotation: *Q*_4_ = −0.02 ± 0.13 Å, *Q*_5_ = 0.02 ± 0.10 Å and *Q*_6_ = −0.00 ± 0.11 Å. In each case, the magnitude of the distortion is zero within the standard deviation, and the value from the average structure presented in Table 2 ▸ also falls within the range of error. This low level of shear generally supports the validity of calculating the *E*_*g*_(*Q*_2_, *Q*_3_) Van Vleck modes along bond directions rather than a Cartesian coordinate system for a system like LaMnO_3_. It is interesting that the standard deviation is higher for *Q*_4_, which quantifies the shear within the plane in which there is orbital ordering.

### 4.3. Effect of pressure on the JT distortion in NaNiO_2_

In recent years, there have been several studies looking at the effect of applied pressure on the JT distortion in crystalline materials (Åsbrink *et al.*, 1999 ▸; Loa *et al.*, 2001 ▸; Choi *et al.*, 2006 ▸; Zhou *et al.*, 2008 ▸, 2011 ▸; Aguado *et al.*, 2012 ▸; Mota *et al.*, 2014 ▸; Caslin *et al.*, 2016 ▸; Zhao *et al.*, 2016 ▸; Collings *et al.*, 2018 ▸; Bhadram *et al.*, 2021 ▸; Lawler *et al.*, 2021 ▸; Scatena *et al.*, 2021 ▸; Ovsyannikov *et al.*, 2021 ▸; Nagle-Cocco *et al.*, 2022 ▸). Most of these have shown that, as a general rule, pressure reduces the magnitude of the JT distortion as a consequence of the elongated bond being more compressible than the shorter bonds.

Zhou *et al.* (2011 ▸) used Van Vleck modes to quantify the effect of pressure on the JT distortion in the corner-sharing perovskite-like compounds LaMnO_3_ and KCuF_3_. While the application of pressure reduces the magnitude of the distortion, as quantified using ρ_0_ [equation (11)], they argue that it does not change the orbital mixing ϕ [equation (12)]. KCuF_3_ has similar orbital ordering to LaMnO_3_, except the degeneracy is due to the *d* ^9^ hole rather than an electron. The variable-pressure crystal structures for KCuF_3_ are available from the ICSD (catalogue codes 182849–182857) and are utilized here.

**Figure 7:**
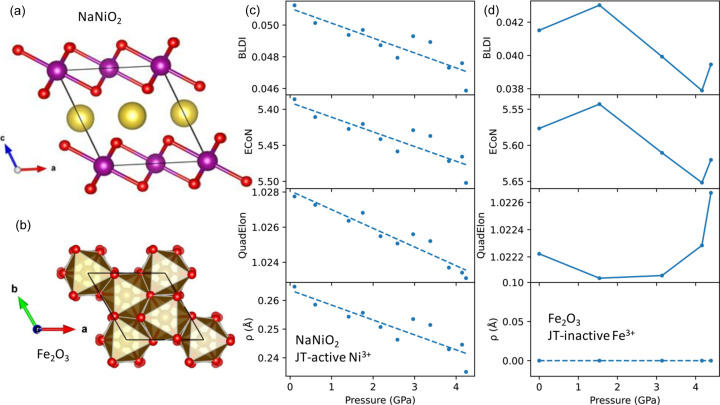
The crystal structures of (*a*) JT-active *C*2/*m* NaNiO_2_ and (*b*) inactive 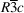 Fe_2_O_3_. (*c*) and (*d*) Comparisons of various metrics for quantifying the degree of JT distortion as a function of pressure, for NiO_6_ octahedra in NaNiO_2_ and FeO_6_ octahedra in Fe_2_O_3_, respectively. The parameters subject to comparison are the magnitude ρ_0_, the bond-length distortion index, the effective coordination number and quadratic elongation. Dashed lines indicate a linear fit to the data, whereas solid lines connect data points.

We previously studied the effect of pressure on the JT distortion in NaNiO_2_ (Nagle-Cocco *et al.*, 2022 ▸) by performing Rietveld refinement of neutron diffraction data from the PEARL instrument (Bull *et al.*, 2016 ▸) at the ISIS Neutron and Muon Source (Oxfordshire, UK). However, we did not utilize the Van Vleck distortion modes, instead quantifying the JT distortion using the bond-length distortion index (Baur, 1974 ▸) and the effective coordination number (Hoppe, 1979 ▸). In that study, we found no deviation from the ambient-pressure space group *C*2/*m* (Dick *et al.*, 1997 ▸; Sofin & Jansen, 2005 ▸), shown in Fig. 7 ▸(*a*), for all pressure points at room temperature up to ∼4.5 GPa. This space group permits only four short (long) and two long (short) bonds or six equal bond lengths, depending on the angle β, and so throughout the measured pressure range there is no *Q*_2_ character to the JT distortion, consistent with the principle that hydrostatic pressure does not change orbital mixing (Zhou *et al.*, 2011 ▸).

Here, we perform a fresh analysis of the variable-pressure octahedral behaviour as a function of pressure at room temperature in NaNiO_2_ in terms of the *E*_*g*_(*Q*_2_, *Q*_3_) Van Vleck distortion modes. For a reference we sought a material that does not exhibit a first-order JT distortion but does exhibit bond-length distortion; for this purpose, we selected Fe_2_O_3_, the pressure dependence of which was previously studied by Finger & Hazen (1980 ▸) and which exhibits bond-length distortion due to its face- and edge-sharing octahedral connectivity. Fe_2_O_3_ contains high-spin *d* ^5^ Fe^3+^ cations within octahedra which interact via both face- and edge-sharing interactions. Note that Fe_2_O_3_ probably exhibits some very subtle pseudo-JT distortion (related to, but distinct from, the first JT effect discussed here) on account of the Fe^3+^ ions (Cumby & Attfield, 2017 ▸; Bersuker & Polinger, 2020 ▸), but this does not impact the discussion in any meaningful way.

In Fig. 7 ▸(*c*) we compare (for NaNiO_2_) ρ_0_ with three other parameters (bond-length distortion index, quadratic elongation and effective coordination number) which are often used to parameterize the magnitude of the JT distortion. The trend for each is near identical, although the magnitudes differ greatly, indicating that each is a reasonable parameter for quantifying the magnitude of the JT distortion. This can be compared with Fig. 7 ▸(*d*), which shows the same parameters for the JT-undistorted FeO_6_ octahedra in Fe_2_O_3_; it can be seen that ρ_0_ remains approximately at zero throughout the measured pressure range, despite a high level of bond-length distortion as represented by the bond-length distortion index, effective coordination number and quadratic elongation (a similar plot for KCuF_3_ can be seen in Fig. S3). This means that, while these parameters are valid for quantifying the magnitude of the JT distortion, they are also sensitive to other kinds of distortion. ρ_0_ is calculated using *Q*_2_ and *Q*_3_ which have *E*_*g*_ symmetry, so ρ_0_ will only be non-zero for a distortion with *E*_*g*_ symmetry. Thus, it is arguably the ideal choice for parameterizing the magnitude of this type of JT distortion. However, while ρ_0_ is more reliable than the other parameters shown in Figs. 7 ▸(*c*) and 7 ▸(*d*) for demonstrating the presence of JT distortion, it is not always strictly zero for a JT-inactive octahedron as it will have a non-zero value if the octahedron is distorted with an *e*_*g*_ symmetry. For example, the NaO_6_ octahedron in *C*2/*m* NaNiO_2_ has the same symmetry as the NiO_6_ octahedron and so exhibits a value of ρ_0_ between 0.065 and 0.05 within the studied pressure range (Fig. S4), and JT-inactive FeO_6_ octahedra in *R*FeO_3_ perovskites have non-zero ρ_0_ due to the *E*_*g*_ symmetry of the distorted octahedra, as shown by Zhou & Goodenough (2008*a* ▸).

**Figure 8:**
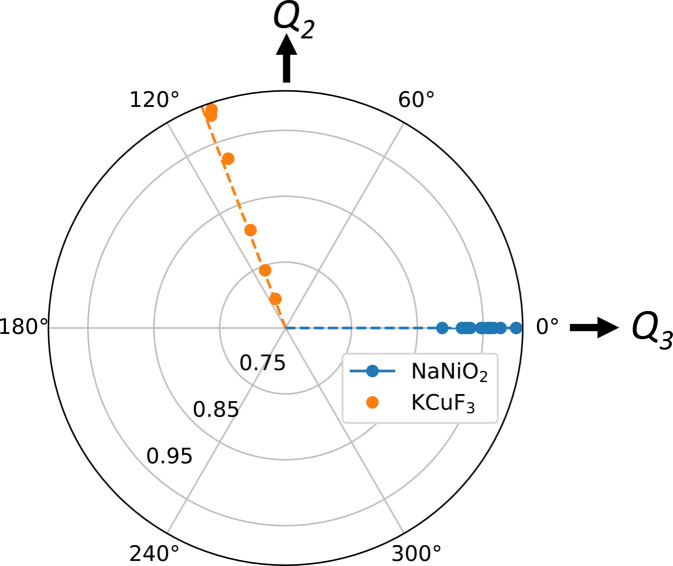
An *E*_*g*_(*Q*_2_, *Q*_3_) radial plot comparing the pressure dependence of the *M*O_6_ (*M* = Ni, Cu) octahedra for KCuF_3_ and NaNiO_2_ between 0 and 5 GPa, where ρ_0_ is normalized to the value at the lowest measured pressure and the dashed lines represent the average ϕ for each material within this pressure range.

Fig. 8 ▸ shows a polar plot for the behaviour of NaNiO_2_ and KCuF_3_ in the range 0–5 GPa (the measured range for NaNiO_2_). It can be seen that within this pressure range, the magnitude of the JT distortion decreases far more for KCuF_3_ than for NaNiO_2_; this reflects the fact that KCuF_3_ is more compressible, with a bulk modulus of 57 (1) GPa (Zhou *et al.*, 2011 ▸) in contrast to 121 (2) GPa for NaNiO_2_ (Nagle-Cocco *et al.*, 2022 ▸), as obtained by a fit to the third-order Birch–Murnaghan equation of state (Birch, 1947 ▸). Within this pressure range we see that ϕ does not change with pressure for either material and that this property is true regardless of whether ϕ is or is not a special angle (as in Table 1 ▸), consistent with the interpretation of Zhou *et al.* (2011 ▸).

**Figure 9:**
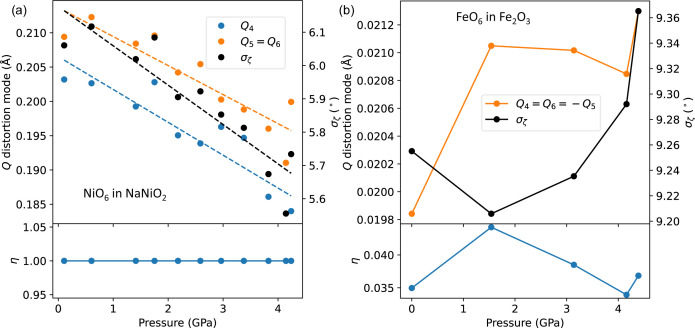
The pressure dependence of the shear and angular distortion in (*a*) JT-distorted NiO_6_ octahedra in NaNiO_2_ and (*b*) JT-undistorted FeO_6_ octahedra in Fe_2_O_3_. Shear distortion is represented with the *Q*_4_, *Q*_5_ and *Q*_6_ modes for the octahedra, and angular distortion is represented by bond-angle variance. Dashed lines indicate a fitted straight line to the data, whereas solid lines are plotted from point to point. η is the angular shear fraction defined in equation (22). Note that for the NiO_6_ octahedra, *Q*_5_ = *Q*_6_, whereas for FeO_6_ octahedra, *Q*_4_ = *Q*_6_ = −*Q*_5_. For Fe_2_O_3_, the average position of the O ligands was taken as the centre of the octahedron.

Finally, in the previous study (Nagle-Cocco *et al.*, 2022 ▸) we showed, using specific O—Ni—O bond angles, that pressure reduces the angular distortion for NaNiO_2_. Here, we show that pressure also reduces the related shear distortion in NaNiO_2_. This is demonstrated in Fig. 9 ▸ where we plot the octahedral shear *Q*_4_, *Q*_5_ and *Q*_6_ modes for NaNiO_2_ and Fe_2_O_3_ against the bond-angle standard deviation σ_ζ_, defined in equation (13). Unlike the AlO_6_ octahedra in LaAlO_3_ (Fig. 5 ▸), for NiO_6_ octahedra in NaNiO_2_ there is no perfect correlation between the shear modes and angular distortion despite η ≃ 1, because there is more than one independent shear mode, but we can see that shear distortion and angular distortion are still highly correlated. However, for Fe_2_O_3_ the shear fraction η ≪ 1 and there is no correlation between the shear distortion modes and angular distortion. This difference in behaviour probably arises because the main driver of the change is a continuous decrease in the JT distortion in NaNiO_2_, while in Fe_2_O_3_ the positions of the oxygen anions are determined by the reduced degrees of freedom arising from trying to satisfy multiple face- and edge-sharing interactions. This result could only be achieved by calculating the Van Vleck modes in a Cartesian coordinate system as outlined in this paper, as opposed to calculating the distortion modes along bond directions, indicating the relevance of calculating the Van Vleck modes in this way and of the shear fraction η we propose in this work.

## 5. Conclusion

We have presented *VanVleckCalculator*, a code package written in Python 3 for the calculation of octahedral Van Vleck distortion modes. These modes are particularly important for understanding the behaviour of the JT distortion, and we have shown that the parameter ρ_0_ (which is based on the Van Vleck *Q*_2_ and *Q*_3_ modes) is a more reliable way of quantifying the JT distortion than other oft-used parameters such as the bond-length distortion index.

We have shown the importance of using a Cartesian set of coordinates for this calculation, instead of calculating the modes along bond directions as is often done in the literature. This is because calculating the Van Vleck distortion modes along bond directions relies on the assumption that there is no angular distortion or octahedral shear, which is often a false assumption and artificially constrains the *Q*_4_, *Q*_5_ and *Q*_6_ modes to be zero. We have shown that there is value in calculating these latter modes, for instance in understanding the effect of octahedral tilting on octahedra in perovskite-like materials. These shear modes will also be useful for parameterizing the JT effect when the degeneracy occurs in the *t*_2*g*_ orbitals and results in a trigonal distortion, because their symmetry matches the distortion.

We have also shown that octahedral shear correlates with angular distortion for materials under the influence of tuning parameters such as pressure or temperature where there is a continuously varying distortion, such as octahedral tilting (as in LaAlO_3_) or first-order JT distortion (as in NaNiO_2_). However, there is no correlation when the distortion is caused by competing interactions due to face- or edge-sharing octahedra (as in Fe_2_O_3_). We propose a new parameter, the shear fraction η [defined in equation (22)], which can be used to predict whether there will be correlation between octahedral shear modes and angular distortion.

## 6. Related literature

For further literature related to the supporting information, see Halasyamani (2004 ▸), Koçer *et al.* (2019 ▸) and Swanson & Peterson (1980 ▸).

## Supplementary Material

Additional information. DOI: 10.1107/S1600576723009925/oc5030sup1.pdf

## Figures and Tables

**Table 1:** The special angles in the *Q*_2_–*Q*_3_ phase space (Fig. 3 ▸) as a function of 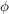 = arctan(*Q*_2_/*Q*_3_), with the associated singly occupied *e*_*g*_ orbital, for *d* ^4^ and low-spin *d* ^7^ octahedral complexes Note that for angles which are not special angles there will be mixing of the orbital states of the nearest two special angles.

ϕ (°)	Ψ(ϕ)
0	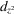
60	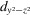
120	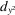
180	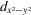
240	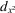
300	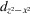

**Table 2:** A comparison between calculated ϕ, ρ_0_, *Q*_2_, *Q*_3_, *Q*_4_, *Q*_5_, *Q*_6_ and η values for NaNiO_2_ and LaMnO_3_ at room temperature, calculated using orthogonal axes as described in this report (‘Cartesian’ method) and alternatively by ignoring angular distortion and calculating Van Vleck modes along bond lengths [‘Kanamori’ method, so named because the equations were originated by Kanamori (1960 ▸)] The centre of the octahedron is taken as the central ion position. Values were calculated using crystal structures reported in the ICSD. To demonstrate the difference in angular distortion, the bond-angle variance BAV [defined in equation (13)] is also tabulated. The BAV is rounded to the third significant figure; *Q* modes and related parameters are rounded to the fourth decimal place.

	NaNiO_2_		LaMnO_3_	
	Kanamori	Cartesian	Kanamori	Cartesian
ICSD code	415072		50334	
Reference	Sofin & Jansen (2005 ▸)		Rodriguez-Carvajal *et al.* (1998 ▸)	
Octahedron	NiO_6_		MnO_6_	
JT-active	Yes		Yes	
Connectivity	Edge		Corner	
BAV (°^2^)	35.2		0.45	

*Q*_2_ (Å)	0.0000	0.0000	0.2745	0.2745
*Q*_3_ (Å)	0.2834	0.2833	−0.0860	−0.0860
*Q*_4_ (Å)	0	0.2078	0	0.0130
*Q*_5_ (Å)	0	0.2001	0	0.0114
*Q*_6_ (Å)	0	0.2001	0	0.0361
ϕ (°)	0.0000†	0.0000	107.3929	107.4034
ρ_0_ (Å)	0.2834	0.2833	0.2877	0.2876
Δ_shear_ (Å)	N/A	0.3534	N/A	0.0389
Δ_anti-shear_ (Å)	N/A	0	N/A	0
η	N/A	1.0	N/A	1.0

† Note that ϕ = 0° is equivalent to 120 or 240°.

## Footnotes

1 In the low-spin case, *t*_2*g*_ orbitals fill fully before *e*_*g*_ orbitals gain electrons; in the high-spin case, once the *t*_2*g*_ orbitals are singly occupied, the next two electrons will populate the *e*_*g*_ orbitals.

2 For a set of three orthogonal vectors chosen as the axes, the choice to assign each to *x*, *y* or *z* will not affect the value of ρ_0_, but will affect the value of  = arctan(*Q*_2_/*Q*_3_) by an integer multiple of 120°, plus a reflection about the nearest special angle (see Table 1 ▸) if there is *Q*_2_–*Q*_3_ mixing.

3 This is because rotation operations do not commute and so a single rotation about each axis is unlikely to result in the defined axes being superimposed over the basis vectors.

## References

[bb1] Aguado, F., Rodríguez, F., Valiente, R., Itiè, J.-P. & Hanfland, M. (2012). *Phys. Rev. B*, **85**, 100101.

[bb2] Ahmed, M. R. & Gehring, G. A. (2006). *Phys. Rev. B*, **74**, 014420.

[bb3] Ahmed, M. R. & Gehring, G. A. (2009). *Phys. Rev. B*, **79**, 174106.

[bb4] Alonso, J. A., Martínez-Lope, M. J., Casais, M. T. & Fernández-Díaz, M. T. (2000). *Inorg. Chem.* **39**, 917–923. 10.1021/ic990921e 12526369

[bb5] Angel, R. J., Zhao, J. & Ross, N. L. (2005). *Phys. Rev. Lett.* **95**, 025503. 10.1103/PhysRevLett.95.025503 16090697

[bb6] Armstrong, A. R. & Bruce, P. G. (1996). *Nature*, **381**, 499–500.

[bb7] Åsbrink, S., Waśkowska, A., Gerward, L., Staun Olsen, J. & Talik, E. (1999). *Phys. Rev. B*, **60**, 12651–12656.

[bb8] Bacci, M., Ranfagni, A., Cetica, M. & Viliani, G. (1975). *Phys. Rev. B*, **12**, 5907–5911.

[bb9] Baur, W. H. (1974). *Acta Cryst.* B**30**, 1195–1215.

[bb10] Bersuker, I. B. & Polinger, V. (2020). *Condens. Matter*, **5**, 68.

[bb11] Bhadram, V. S., Joseph, B., Delmonte, D., Gilioli, E., Baptiste, B., Le Godec, Y., Lobo, R. P. S. M. & Gauzzi, A. (2021). *Phys. Rev. Mater.* **5**, 104411.

[bb12] Birch, F. (1947). *Phys. Rev.* **71**, 809–824.

[bb13] Bleaney, B. & Bowers, K. D. (1952). *Proc. Phys. Soc. A*, **65**, 667–668.

[bb14] Bocharov, D., Krack, M., Rafalskij, Y., Kuzmin, A. & Purans, J. (2020). *Comput. Mater. Sci.* **171**, 109198.

[bb15] Bull, C. L., Funnell, N. P., Tucker, M. G., Hull, S., Francis, D. J. & Marshall, W. G. (2016). *High. Press. Res.* **36**, 493–511.

[bb16] Capone, M., Feinberg, D. & Grilli, M. (2000). *Eur. Phys. J. B*, **17**, 103–109.

[bb17] Caslin, K., Kremer, R. K., Razavi, F. S., Hanfland, M., Syassen, K., Gordon, E. E. & Whangbo, M.-H. (2016). *Phys. Rev. B*, **93**, 022301.

[bb18] Castillo-Martínez, E., Bieringer, M., Shafi, S. P., Cranswick, L. M. D. & Alario-Franco, M. Á. (2011). *J. Am. Chem. Soc.* **133**, 8552–8563. 10.1021/ja109376s 21574562

[bb19] Chatterji, T., Fauth, F., Ouladdiaf, B., Mandal, P. & Ghosh, B. (2003). *Phys. Rev. B*, **68**, 052406.

[bb20] Chiang, F.-K., Chu, M.-W., Chou, F. C., Jeng, H. T., Sheu, H. S., Chen, F. R. & Chen, C. H. (2011). *Phys. Rev. B*, **83**, 245105.

[bb21] Child, M. S. & Roach, A. C. (1965). *Mol. Phys.* **9**, 281–285.

[bb22] Choi, H. C., Shim, J. H. & Min, B. I. (2006). *Phys. Rev. B*, **74**, 172103.

[bb23] Collings, I. E., Bykov, M., Bykova, E., Hanfland, M., van Smaalen, S., Dubrovinsky, L. & Dubrovinskaia, N. (2018). *CrystEngComm*, **20**, 3512–3521. 10.1039/c8cp03761b 30221645

[bb24] Cumby, J. & Attfield, J. P. (2017). *Nat. Commun.* **8**, 14235. 10.1038/ncomms14235 PMC5296646 28146146

[bb25] Cussen, E. J., Rosseinsky, M. J., Battle, P. D., Burley, J. C., Spring, L. E., Vente, J. F., Blundell, S. J., Coldea, A. I. & Singleton, J. (2001). *J. Am. Chem. Soc.* **123**, 1111–1122. 10.1021/ja003139i 11456664

[bb26] Darlington, C. N. W. (1996). *Phys. Status Solidi A*, **155**, 31–42.

[bb27] Dick, S., Müller, M., Preissinger, F. & Zeiske, T. (1997). *Powder Diffr.* **12**, 239–241.

[bb28] Dong, S., Zhang, Q., Yunoki, S., Liu, J.-M. & Dagotto, E. (2012). *Phys. Rev. B*, **86**, 205121.

[bb29] Fedorova, N. S., Ederer, C., Spaldin, N. A. & Scaramucci, A. (2015). *Phys. Rev. B*, **91**, 165122.

[bb30] Fedorova, N. S., Windsor, Y. W., Findler, C., Ramakrishnan, M., Bortis, A., Rettig, L., Shimamoto, K., Bothschafter, E. M., Porer, M., Esposito, V., Hu, Y., Alberca, A., Lippert, T., Schneider, C. W., Staub, U. & Spaldin, N. A. (2018). *Phys. Rev. Mater.* **2**, 104414.

[bb31] Fil, D. V., Tokar, O. I., Shelankov, A. L. & Weber, W. (1992). *Phys. Rev. B*, **45**, 5633–5640. 10.1103/physrevb.45.5633 10000282

[bb32] Finger, L. W. & Hazen, R. M. (1980). *J. Appl. Phys.* **51**, 5362–5367.

[bb33] Franchini, C., Archer, T., He, J., Chen, X.-Q., Filippetti, A. & Sanvito, S. (2011). *Phys. Rev. B*, **83**, 220402.

[bb34] García, J., Subías, G., Sánchez, M. C. & Blasco, J. (2005). *Phys. Scr.* **2005**(T115), 594.

[bb35] Genreith-Schriever, A. R., Alexiu, A., Phillips, G. S., Coates, C. S., Nagle-Cocco, L. A. V., Bocarsly, J. D., Sayed, F. N., Dutton, S. E. & Grey, C. P. (2023). *ChemRxiv*, https://doi.org/10.26434/chemrxiv-2023-q3cmz.

[bb36] Goodenough, J. B. (1998). *Annu. Rev. Mater. Sci.* **28**, 1–27.

[bb37] Goodenough, J. B., Wold, A., Arnott, R. J. & Menyuk, N. J. P. R. (1961). *Phys. Rev.* **124**, 373–384.

[bb38] Goodwin, A. L. (2017). *Orbital (Dis)order: A Tale of Two Temperatures.* Goodwin Group Inorganic Chemistry Laboratory University of Oxford, UK. https://goodwingroupox.uk/etc/project-two-xt38h.

[bb39] Halasyamani, P. S. (2004). *Chem. Mater.* **16**, 3586–3592.

[bb40] Halcrow, M. A. (2009). *Coord. Chem. Rev.* **253**, 2493–2514.

[bb41] Harris, C. R., Millman, K. J., van der Walt, S. J., Gommers, R., Virtanen, P., Cournapeau, D., Wieser, E., Taylor, J., Berg, S., Smith, N. J., Kern, R., Picus, M., Hoyer, S., van Kerkwijk, M. H., Brett, M., Haldane, A., del Río, J. F., Wiebe, M., Peterson, P., Gérard-Marchant, P., Sheppard, K., Reddy, T., Weckesser, W., Abbasi, H., Gohlke, C. & Oliphant, T. E. (2020). *Nature*, **585**, 357–362. 10.1038/s41586-020-2649-2 PMC7759461 32939066

[bb42] Hayward, S. A., Morrison, F. D., Redfern, S. A. T., Salje, E. K. H., Scott, J. F., Knight, K. S., Tarantino, S., Glazer, A. M., Shuvaeva, V., Daniel, P., Zhang, M. & Carpenter, M. A. (2005). *Phys. Rev. B*, **72**, 054110.

[bb43] Holland, J. M., McAllister, J. A., Kilner, C. A., Thornton-Pett, M., Bridgeman, A. J. & Halcrow, M. A. (2002). *J. Chem. Soc. Dalton Trans.* pp. 548–554.

[bb44] Hoppe, R. (1979). *Z. Kristallogr. Cryst. Mater.* **150**, 23–52.

[bb45] Hunter, J. D. (2007). *Comput. Sci. Eng.* **9**, 90–95.

[bb46] Jahn, H. A. & Teller, E. (1937). *Proc. R. Soc. London Ser. A*, **161**, 220–235.

[bb47] Jansen, M. & Hoppe, R. (1973). *Z. Anorg. Allge Chem.* **399**, 163–169.

[bb48] Ji, C., Wang, Y., Guo, B., Shen, X., Luo, Q., Wang, J., Meng, X., Zhang, J., Lu, X. & Zhu, J. (2019). *Phys. Rev. B*, **100**, 174417.

[bb49] Kanamori, J. (1960). *J. Appl. Phys.* **31**, S14–S23.

[bb50] Keller, H., Bussmann-Holder, A. & Müller, K. A. (2008). *Mater. Today*, **11**, 38–46.

[bb51] Khomskii, D. I. & Streltsov, S. V. (2021). *Chem. Rev.* **121**, 2992–3030. 10.1021/acs.chemrev.0c00579 33314912

[bb52] Kimber, S. A. J. (2012). *J. Phys. Condens. Matter*, **24**, 186002. 10.1088/0953-8984/24/18/186002 22499064

[bb53] King, G. & Woodward, P. M. (2010). *J. Mater. Chem.* **20**, 5785–5796.

[bb54] Koçer, C. P., Griffith, K. J., Grey, C. P. & Morris, A. J. (2019). *J. Am. Chem. Soc.* **141**, 15121–15134. 10.1021/jacs.9b06316 31448601

[bb55] Koskelo, E. C., Kelly, N. D., Nagle-Cocco, L. A. V., Bocarsly, J. D., Mukherjee, P., Liu, C., Zhang, Q. & Dutton, S. E. (2023). *Inorg. Chem.* **62**, 10317–10328. 10.1021/acs.inorgchem.3c01137 PMC10324300 37326623

[bb56] Kyono, A., Gramsch, S. A., Nakamoto, Y., Sakata, M., Kato, M., Tamura, T. & Yamanaka, T. (2015). *Am. Mineral.* **100**, 1752–1761.

[bb57] Lawler, K. V., Smith, D., Evans, S. R., dos Santos, A. M., Molaison, J. J., Bos, J. G., Mutka, H., Henry, P. F., Argyriou, D. N., Salamat, A. & Kimber, S. A. J. (2021). *Inorg. Chem.* **60**, 6004–6015. 10.1021/acs.inorgchem.1c00435 33788545

[bb58] Lindner, F. P., Aichhorn, M. & Banerjee, H. (2022). *arXiv*:2212.01090.

[bb59] Loa, I., Adler, P., Grzechnik, A., Syassen, K., Schwarz, U., Hanfland, M., Rozenberg, G. K., Gorodetsky, P. & Pasternak, M. P. (2001). *Phys. Rev. Lett.* **87**, 125501. 10.1103/PhysRevLett.87.125501 11580518

[bb60] Lufaso, M. W. & Woodward, P. M. (2004). *Acta Cryst.* B**60**, 10–20. 10.1107/S0108768103026661 14734840

[bb61] Marshall, L. G., Zhou, J.-S., Zhang, J., Han, J., Vogel, S. C., Yu, X., Zhao, Y., Fernández-Díaz, M.-T., Cheng, J. & Goodenough, J. B. (2013). *Phys. Rev. B*, **87**, 014109.

[bb62] Mikheykin, A. S., Torgashev, V. I., Yuzyuk, Y. I., Bush, A. A., Talanov, V. M., Cervellino, A. & Dmitriev, V. P. (2015). *J. Phys. Chem. Solids*, **86**, 42–48.

[bb63] Millis, A. J., Shraiman, B. I. & Mueller, R. (1996). *Phys. Rev. Lett.* **77**, 175–178. 10.1103/PhysRevLett.77.175 10061800

[bb64] Momma, K. & Izumi, F. (2011). *J. Appl. Cryst.* **44**, 1272–1276.

[bb65] Moron, M. C., Palacio, F. & Rodríguez-Carvajal, J. (1993). *J. Phys. Condens. Matter*, **5**, 4909–4928.

[bb66] Mota, D. A., Almeida, A., Rodrigues, V. H., Costa, M. M. R., Tavares, P., Bouvier, P., Guennou, M., Kreisel, J. & Moreira, J. A. (2014). *Phys. Rev. B*, **90**, 054104.

[bb67] Nagle-Cocco, L. A. V. (2023). *VanVleckCalculator*, https://github.com/lnaglecocco/VanVleckCalculator.

[bb68] Nagle-Cocco, L. A. V., Bull, C. L., Ridley, C. J. & Dutton, S. E. (2022). *Inorg. Chem.* **61**, 4312–4321. 10.1021/acs.inorgchem.1c03345 PMC9098164 35238545

[bb69] Ong, S. P., Richards, W. D., Jain, A., Hautier, G., Kocher, M., Cholia, S., Gunter, D., Chevrier, V. L., Persson, K. A. & Ceder, G. (2013). *Comput. Mater. Sci.* **68**, 314–319.

[bb70] Ovsyannikov, S. V., Aslandukova, A. A., Aslandukov, A., Chariton, S., Tsirlin, A. A., Korobeynikov, I. V., Morozova, N. V., Fedotenko, T., Khandarkhaeva, S. & Dubrovinsky, L. (2021). *Inorg. Chem.* **60**, 13440–13452. 10.1021/acs.inorgchem.1c01782 34492760

[bb71] Peterse, W. J. A. M. & Palm, J. H. (1966). *Acta Cryst.* **20**, 147–150.

[bb72] Pughe, C. E., Mustonen, O. H. J., Gibbs, A. S., Lee, S., Stewart, R., Gade, B., Wang, C., Luetkens, H., Foster, A., Coomer, F. C., Takagi, H. & Cussen, E. J. (2023). *Chem. Mater.* **35**, 2752–2761. 10.1021/acs.chemmater.2c02939 PMC10100530 37063596

[bb73] Qiu, X., Proffen, T., Mitchell, J. F. & Billinge, S. J. L. (2005). *Phys. Rev. Lett.* **94**, 177203. 10.1103/PhysRevLett.94.177203 15904332

[bb74] Ren, W.-N., Jin, K., Guo, E.-J., Ge, C., Wang, C., Xu, X., Yao, H., Jiang, L. & Yang, G. (2021). *Phys. Rev. B*, **104**, 174428.

[bb75] Rietveld, H. M. (1969). *J. Appl. Cryst.* **2**, 65–71.

[bb76] Robinson, K., Gibbs, G. V. & Ribbe, P. H. (1971). *Science*, **172**, 567–570. 10.1126/science.172.3983.567 17802221

[bb77] Rodríguez-Carvajal, J., Hennion, M., Moussa, F., Moudden, A. H., Pinsard, L. & Revcolevschi, A. J. (1998). *Phys. Rev. B*, **57**, R3189–R3192. 10.1103/physrevb.54.15149 9985575

[bb78] Sarkar, A., Djenadic, R., Wang, D., Hein, C., Kautenburger, R., Clemens, O. & Hahn, H. (2018). *J. Eur. Ceram. Soc.* **38**, 2318–2327.

[bb79] Scatena, R., Andrzejewski, M., Johnson, R. D. & Macchi, P. (2021). *J. Mater. Chem. C.* **9**, 8051–8056. 10.1039/d1tc01966j PMC8246535 34277008

[bb80] Schmitt, M. M., Zhang, Y., Mercy, A. & Ghosez, P. (2020). *Phys. Rev. B*, **101**, 214304.

[bb81] Schofield, P. F., Knight, K. S., Redfern, S. A. T. & Cressey, G. (1997). *Acta Cryst.* B**53**, 102–112.

[bb82] Shirako, Y., Shi, Y. G., Aimi, A., Mori, D., Kojitani, H., Yamaura, K., Inaguma, Y. & Akaogi, M. (2012). *J. Solid State Chem.* **191**, 167–174.

[bb83] Snamina, M. & Oleś, A. M. (2016). *Phys. Rev. B*, **94**, 214426.

[bb84] Sofin, M. & Jansen, M. (2005). *Z. Naturforsch. Teil B*, **60**, 701–704.

[bb85] Souza, R. A., Ramos, A. Y., Tolentino, H. C. N. & Granado, E. (2005). *Phys. Scr.* **2005**(T115), 428.

[bb86] Streltsov, S. V., Temnikov, F. V., Kugel, K. I. & Khomskii, D. I. (2022). *Phys. Rev. B*, **105**, 205142.

[bb87] Swanson, D. K. & Peterson, R. C. (1980). *Can. Mineral.* **18**, 153–156.

[bb88] Tachibana, M., Shimoyama, T., Kawaji, H., Atake, T. & Takayama-Muromachi, E. (2007). *Phys. Rev. B*, **75**, 144425.

[bb89] Teyssier, J., Giannini, E., Stucky, A., Černý, R., Eremin, M. & van der Marel, D. (2016). *Phys. Rev. B*, **93**, 125138.

[bb90] Thygesen, P. M. M., Young, C. A., Beake, E. O. R., Romero, F. D., Connor, L. D., Proffen, T. E., Phillips, A. E., Tucker, M. G., Hayward, M. A., Keen, D. A. & Goodwin, A. L. (2017). *Phys. Rev. B*, **95**, 174107.

[bb91] Tucker, M. G., Keen, D. A., Dove, M. T., Goodwin, A. L. & Hui, Q. (2007). *J. Phys. Condens. Matter*, **19**, 335218. 10.1088/0953-8984/19/33/335218 21694141

[bb92] Tummers, B. (2006). *DataThief III*, https://datathief.org/.

[bb93] Van Rossum, G. & Drake, F. L. (2009). *Python 3 Reference Manual.* Scotts Valley: CreateSpace.

[bb94] Van Vleck, J. H. (1939). *J. Chem. Phys.* **7**, 72–84.

[bb95] Wang, J., Wang, Z. D., Zhang, W. & Xing, D. Y. (2002*a*). *Phys. Rev. B*, **66**, 064406.

[bb96] Wang, J., Zhang, W. & Xing, D. Y. (2002*b*). *Phys. Rev. B*, **66**, 052410.

[bb97] Woodward, P. M. (1997). *Acta Cryst.* B**53**, 32–43.

[bb98] Xu, L., Meng, J., Liu, Q., Meng, J., Liu, X. & Zhang, H. (2020). *Phys. Chem. Chem. Phys.* **22**, 4905–4915. 10.1039/c9cp06275k 32073064

[bb99] Zhao, Y., Yang, W., Li, N., Li, Y., Tang, R., Li, H., Zhu, H., Zhu, P. & Wang, X. (2016). *J. Phys. Chem. C*, **120**, 9436–9442.

[bb100] Zhou, J.-S., Alonso, J. A., Han, J. T., Fernández-Díaz, M., Cheng, J.-G. & Goodenough, J. B. (2011). *J. Fluor. Chem.* **132**, 1117–1121.

[bb101] Zhou, J.-S. & Goodenough, J. B. (2006). *Phys. Rev. Lett.* **96**, 247202. 10.1103/PhysRevLett.96.247202 16907275

[bb102] Zhou, J.-S. & Goodenough, J. B. (2008*a*). *Phys. Rev. B*, **77**, 132104.

[bb103] Zhou, J.-S. & Goodenough, J. B. (2008*b*). *Phys. Rev. B*, **77**, 172409.

[bb104] Zhou, J.-S., Uwatoko, Y., Matsubayashi, K. & Goodenough, J. B. (2008). *Phys. Rev. B*, **78**, 220402.

