# Supplementary material for: Van Vleck analysis of angularly distorted octahedra using VanVleckCalculator
Source: J Appl Crystallogr. 2024 Feb 1;57(Pt 1):20–33. doi: 10.1107/S1600576723009925 (PMC10840309; doi:10.1107/S1600576723009925)
Supplement: Supplementary file 1 [file j-57-00020-sup1.pdf]

JOURNAL OF
APPLIED
CRYSTALLOGRAPHY

Volume 57 (2024)

Supporting information for article:

Van Vleck analysis of angularly distorted octahedra using *VanVleckCalculator*

Liam. A. V. Nagle-Cocco and Siân E. Dutton

SUPPLEMENTARY INFORMATION

Contents

List of figures 3

List of tables 3

VanVleckCalculator: how to use 4

Necessary packages 4

Importing VanVleckCalculator 4

Creating a PyMatGen structure object 4

Initialising an instance of the Octahedron class 6

Octahedral rotation algorithm 7

Performing calculations: van Vleck modes 7

Performing calculations: angular shear 11

Performing calculations: unrelated to the van Vleck modes 12

Van Vleck Q_3 mode along different axes 15

Octahedral shear mode distribution for $LaMnO_3$ 16

Pressure-dependent distortion of CuF_6 octahedra in $KCuF_3$ 17

Pressure-dependent distortion of NaO_6 octahedra in $NaNiO_2$ 18

Angular shear with pressure and temperature 20

Graphical explanation of the shear angular modes 22

Tabulated data from the main paper 23

List of Figures

S1 Flow chart explaining the octahedron rotation algorithm in VanVleckCalculator 8

S2 Distribution of shear mode values for MnO_6 octahedra in supercell $LaMnO_3$ 16

S3 Pressure-dependence of bond length distortion parameters for CuO_6 octahedra in $KCuF_3$ 17

S4 Pressure-dependence of bond length distortion parameters for NaO_6 octahedra in $NaNiO_2$ 18

S5 Pressure-dependence of shear and angular distortion in $NaNiO_2$ and Fe_2O_3 . 20

S6 Temperature-dependence of shear and angular distortion in $LaAlO_3$ 21

S7 Graphical explanation of the shear angular modes 22

List of Tables

S1 Variable-pressure parameters of CuO_6 octahedra in $KCuF_3$ 24

S2 Variable-pressure parameters of NiO_6 octahedra in $NaNiO_2$ 25

S3 Variable-pressure parameters of FeO_6 octahedra in Fe_2O_3 26

S4 Variable-temperature parameters of AlO_6 octahedra in $LaAlO_3$ 27

S5 Variable-temperature lattice parameters of rhombohedral $LaAlO_3$ 36

S6 Variable-temperature lattice parameters of cubic $LaAlO_3$ 40

VanVleckCalculator: how to use

VanVleckCalculator takes an object-oriented approach, defining first an `Octahedron` class and performing all subsequent calculations as methods of this class.

Necessary packages

VanVleckCalculator requires the following packages:

- NumPy[1]
- PyMatGen[2]

Importing VanVleckCalculator

To use VanVleckCalculator, the code must be saved locally. This can be done either by cloning the repository from GitHub or saving the code directly. It can then be accessed in a Python script as follows:

```
import sys
sys.path.insert(1,r"C:\Users\User\Documents\GitHub\VanVleckCalculator\code")
from van_vleck_calculator import *
```

where the file path given as an argument to the `insert()` function should be modified to match the location of VanVleckCalculator saved locally.

Creating a PyMatGen structure object

PyMatGen structure objects can be accessed using the following code:

```
from pymatgen.core.structure import Structure
```

Once this code has been imported, there are several ways to proceed. A `Structure` object can be generated manually, or using a crystal structure file such as a CIF or a CONTCAR. If generating the `Structure` object manually, the `Lattice` class must also be imported:

```
from pymatgen.core.lattice import Lattice
```

Here, an example is given to generate a `Structure` object manually, the α-MnO_2 structure with $I4/m$ space group:

```
latt = Lattice(matrix=([9.85,0,0],[0,9.85,0],[0,0,2.86]))
local_coords = (
    [0.35049,0.16700,0],
    [0.15137,0.19876,0],
    [0.54139,0.16782,0]
)
struc = Structure(lattice=latt,species=["Mn","O","O"],coords=local_coords)
struc = struc.from_spacegroup(
    lattice=latt,
    species=[Mn","O","O"],
    coords=local_coords,
    sg="I4/m"
)
```

Here, using the `Structure()` initialisation method, we generate a cell with $P1$ symmetry containing just 3 sites at the Wyckoff positions. The `from_spacegroup()` method then replicates these atoms based on the space group given with the argument `sg="I4/m"`.

Alternatively, and generally more conveniently, `Structure` objects will be taken from a CIF. An example is given below of how to do this:

```
struc = Structure.from_file("cif_name.cif")
```

Note that PyMatGen will fix sites that are near a high-symmetry position to be at that symmetry position. This is usually useful for ensuring plausible structures, however for analysis of large structures such as the output of molecular dynamics of big box PDF analysis, this is detrimental. Including in the `from_file()` method the argument `frac_tolerance=0` will prevent this from occurring, but note this is only possible with PyMatGen version 2023.01.20 or later.

Initialising an instance of the Octahedron class

To initialise an instance of the `Octahedron` class, the necessary arguments are a PyMatGen `PeriodicSite` object, and a PyMatGen `Structure` object, where the `PeriodicSite` object is contained within the `Structure` object. For the example of α-MnO_2 given in the previous section, this code would generate an `Octahedron` object associated with the MnO_6 octahedra.

```
MnO6 = Octahedron(struc[0],struc)
```

This method will create an `Octahedron` object with the six nearest atoms as ligands, and check that the structure corresponds to that expected for an octahedron.

The `Octahedron` initialisation method can take additional optional arguments:

- `possible_ligands` is an argument which must be a Python list containing strings corresponding to atoms which may function as ligands. For instance, in the example of α-MnO_2, one might use `possible_ligands=["O"]`. The default is to allow any atom within range to be a ligand.

- `forbidden_ligands` is an argument which must be a Python list containing strings corresponding to atoms which may not function as ligands. For instance, in the example of α-MnO_2, one might use `forbidden_ligands=["Mn"]` to ensure that no Mn cations are inadvertently made ligands. This serves the same function as `possible_ligands`. By default, `forbidden_ligands=[]`, i.e. an empty list.

- `ligands_max_distance` is an argument which sets the maximum distance to which ligands may be located, in units of Ångstroms. This parameter defaults to 2.7 Å.

- `ligands_min_distance` is an argument which sets the minimum distance to which ligands may be located, in units of Ångstroms. This parameter defaults to 0.0 Å.

Octahedral rotation algorithm

As described in the main text, VanVleckCalculator uses an algorithm to rotate the octahedron in order to position ligands as close as possible to the axes and thus minimise the adverse effect of angular distortion on the calculation. To aid understanding of this algorithm, Figure S1 shows the process VanVleckCalculator follows in order to achieve this rotation. Here, we assume the user argument `ignore_angular_distortion=False` (note that the arguments are described further in later sections of this SI).

Performing calculations: van Vleck modes

VanVleckCalculator can be used to calculate the van Vleck modes defined in Equations 5 to 10, along with the polar parameters ρ_0 and ϕ (Equations 11and 12). This section will list all the methods which can be used in calculating these parameters. There are also several optional arguments which may be supplied by the user, which will be listed at the end of this subsection.

The methods of the `Octahedron` class which may be called include:

- `calculate_van_vleck_distortion_modes` : this method calculates the van Vleck modes, returning the results in a Python list containing six elements. An example of this code in use:

```
modes = oct1.calculate_van_vleck_distortion_modes()
Q1 = modes[0]
```

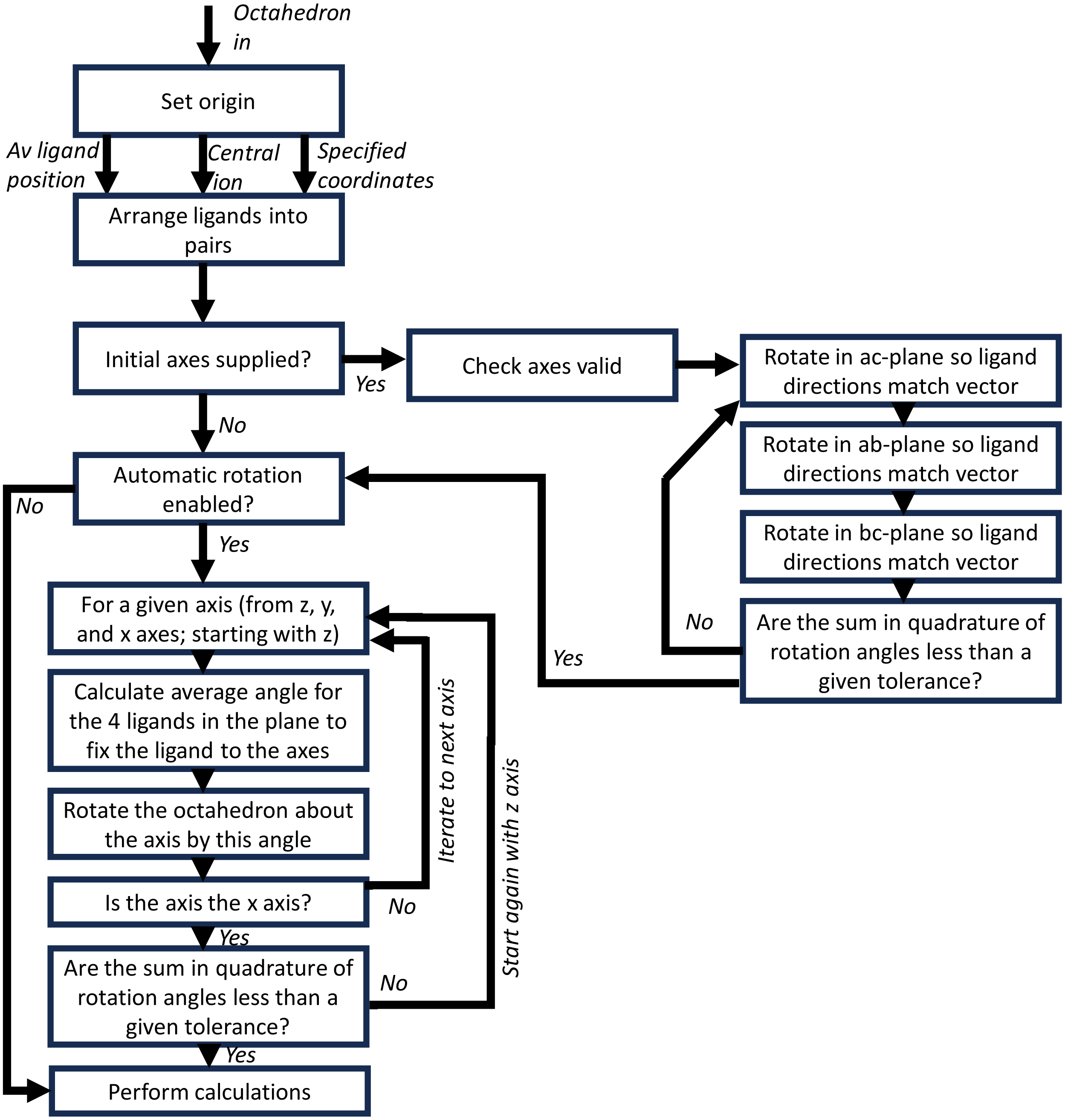

Figure S1: A flow chart explaining the octahedron rotation algorithm in VanVleckCalculator, assuming the user argument ignore_angular_distortion is False.

```
Q2 = modes[1]
Q3 = modes[2]
Q4 = modes[3]
Q5 = modes[4]
Q6 = modes[5]
```

- `calculate_van_vleck_jahn_teller_params` : this method calculates the magnitude and angle in $E_g(Q_2, Q_3)$ phase space, ρ_0, and ϕ respectively, as given in the main text. It returning the results in a Python list containing two elements. An example of this code in use:

```
params = oct1.calculate_van_vleck_jahn_teller_params()
rho = params[0]
phi = params[1]
```

- `calculate_degenerate_Q3_van_vleck_modes` : this method calculates a Python list containing three elements. These are $[Q_3, Q_3', Q_3'']$, where the latter two elements are defined in Equations S1 and S2. It can be executed as follows:

```
Q3_modes = oct1.calculate_degenerate_Q3_van_vleck_modes()
```

- `output_sites_for_van_vleck` : this method returns a Python list containing three elements, representing the three pairs of ligands constituting the vertices of the octahedron. Each of these elements is a list containing two elements, representing one atom each. These final lists contain three coordinates: the x, y, and z coordinates of the given atom. This method may be useful for testing the rotation algorithms of the octahedron. It can be executed as follows:

```
oct1.output_sites_for_van_vleck()
```

- `visualise_sites_for_van_vleck` : this method produces a 3D plot showing the rotated sites relative to the centre of the octahedron. It can be used to ensure the rotation algorithm is producing reasonable results for a given octahedron. It uses Matplotlib.[3] It can be executed as follows:

```
oct1.visualise_sites_for_van_vleck()
```

There are several optional arguments which may be supplied to the above methods, which determine how the rotation algorithm operates and what is considered the "centre" of the octahedron. These optional arguments are:

→ `specified_axes` : *Python list.* A list of three further lists representing vectors. The three vectors must be orthogonal. If this argument is supplied, the automatic octahedral rotation algorithm will not execute, and the octahedron will be rotated such that the given axes are the basis.

→ `starting_axes` : *Python list.* A list of three further lists representing vectors, in the same format as `specified_axes`. If this argument is supplied, before the automatic octahedral rotation algorithm executes, the octahedron will be rotated such that the given axes are the basis. This may be useful for controlling which of the three axes exhibits a tetragonal elongation, or for ensuring that the same axes in all octahedra is used in analysis of many octahedra in a supercell.

→ `octahedral_centre` : *string or Python list.* Defaults to string `"core_atom"` in which case the central atom is taken as the centre of the octahedron. Another option is `"average_ligand_position"` in which case the centre of the octahedron is taken to

be the average of the positions of the six ligands, rather than the core cation. Finally, a list of three floats may be supplied, corresponding to a set of coordinates which will be the centre of the octahedron.

→ `ignore_angular_distortion` : *boolean.* Defaults to False. If True, the code will not rotate the octahedron and will calculate the van Vleck modes using only the bond lengths. This will force the Q_4 to Q_6 modes to be zero.

Performing calculations: angular shear

VanVleckCalculator can be used to calculate the angular shear and anti-shear parameters defined in Equations 14 to 19, the Δ_{shear} and $\Delta_{\text{anti-shear}}$ parameters defined in Equations 20 and 21, and the shear fraction η defined in Equation 22. Here, we give examples for an instance of the `Octahedron` class called `oct_1`.

- `calculate_angular_shear_modes` : this method calculates the angular shear modes, returning them as three elements in a Python list. For example:

```
shear_modes = oct_1.calculate_angular_shear_modes()
```

- `calculate_angular_antishear_modes` : this method calculates the angular anti-shear modes, returning them as three elements in a Python list. For example:

```
antishear_modes = oct_1.calculate_angular_antishear_modes()
```

- `calculate_angular_shear_magnitude` : this method calculates the Δ_{shear} parameter defined in Equations 20. For example:

```
Delta_shear = oct_1.calculate_angular_shear_magnitude()
```

- `calculate_angular_antishear_magnitude` : this method calculates the $\Delta_{\text{anti-shear}}$ parameter defined in Equations 21. For example:

```
Delta_antishear = oct_1.calculate_angular_antishear_magnitude()
```

- `calculate_shear_fraction_angular_distortion` : this method calculates the shear fraction η (defined in Equation 22). For example:

```
eta = oct_1.calculate_shear_fraction_angular_distortion()
```

These three methods can take most of the arguments intended for calculation of the van Vleck modes, i.e. `specified_axes`, `starting_axes`, and `octahedral_centre`. They cannot take `ignore_angular_distortion` as this argument would constrain them to be zero.

Performing calculations: unrelated to the van Vleck modes

VanVleckCalculator can also be used to calculate various parameters unrelated to the van Vleck modes. These include octahedral volume and various distortion parameters. Here, we give examples for an instance of the `Octahedron` class called `oct_1`.

- Octahedral volume is calculated following the method in Ref.[4] The parameter is calculated with the method `calculate_volume` as follows:

```
volume = oct_1.calculate_volume()
```

- The core-ligand bond length for a perfect octahedron with identical volume to `oct1` is calculated as follows:

```
l_0 = oct1.calculate_core_ligand_distance_for_perfect_octahedron()
```

  This parameter is useful for calculating quadratic elongation.

- Bond length distortion index[5] is calculated as follows:

```
BLDI = oct_1.calculate_bond_length_distortion_index()
```

  This parameter has the optional argument `octahedral_centre` which must be a string. It defaults to `"core_atom"`, which corresponds to the parameter as defined in Ref.[5] It can also be set to `"average_ligand_position"`, the bond lengths are taken relative to the average position of the 6 ligands, rather than the core cation.

- Quadratic elongation[6] can be calculated as follows:

```
QuadElon = oct1.calculate_quadratic_elongation()
```

- Effective coordination[7] can be calculated as follows:

```
ECoN = oct1.calculate_effective_coordination_number()
```

- Bond angle variance[5] can be calculated as follows:

```
BAV = oct1.calculate_bond_angle_variance()
```

This method has an optional boolean argument `degrees` which defaults to True. If True, returns a value in degrees squared, otherwise in radians squared.

- Off-centering distance,[8] a metric for quantifying the pseudo Jahn-Teller distortion, can be calculated as follows:

```
OCD = oct1.calculate_off_centering_distance()
```

- Another off-centering metric[9] for quantifying the pseudo Jahn-Teller distortion, can be calculated as follows:

```
OCM = oct1.calculate_off_centering_metric()
```

Van Vleck Q_3 mode along different axes

The van Vleck Q_3 mode represents tetragonal distortion of an octahedron, and was defined in the main text. However this equation assumes that the tetragonal distortion occurs along the c-axis. However, the axis definitions can sometimes be arbitrary and there is a chance the tetragonal distortion may occur along the a- or b-axes. When this occurs, changing the order of the vectors given to the `starting_axes` or `specified_axes` arguments in VanVleckCalculator can change the basis to give the elongation along the c axis.

Alternatively, the tetragonal elongation can be calculated along the alternative axes by these equations:

$$Q_3' = \frac{1}{2}\left[-Q_3 + \sqrt{3}Q_2\right] \tag{S1}$$

$$Q_3'' = \frac{1}{2}\left[-Q_3 - \sqrt{3}Q_2\right] \tag{S2}$$

This makes use of the fact that a change in axes corresponds to a 120° rotation in the $E_g(Q_2, Q_3)$ phase space. To our knowledge, it was first noted in Ref.[10]

Octahedral shear mode distribution for $LaMnO_3$

In the main text, we presented analysis of the MnO_6 octahedra in a supercell of $LaMnO_3$, which was obtained by reverse Monte Carlo analysis of room-temperature pair distribution function data in a previous study. We calculated the Q_4 to Q_6 octahedral shear modes for all octahedra in the supercell, with mean and standard deviations as follows: $Q_4 = -0.02 \pm 0.13$, $Q_5 = 0.02 \pm 0.10$, and $Q_6 = -0.00 \pm 0.11$. The values for all octahedra are shown as histograms in Figure S2, and it can be seen that the distribution is centred on zero, consistent with the absence of octahedral shear except due to local thermal fluctuations.

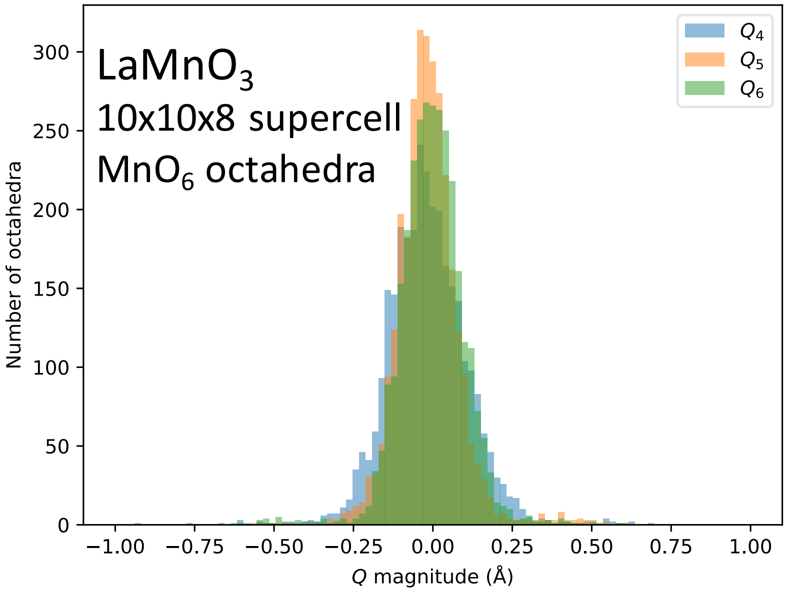

Figure S2: The distribution of the octahedral shear modes Q_4 to Q_6 for the MnO_6 octahedra in a supercell of $LaMnO_3$, as obtained by reverse Monte Carlo analysis of room-temperature pair distribution function data. Supercell CIF obtained from the authors of Ref.[11]

Pressure-dependent distortion of CuF_6 octahedra in $KCuF_3$

In the main text of the manuscript, we show the pressure-dependence of the NiO_6 and FeO_6 octahedral distortion in Jahn-Teller-distorted $NaNiO_2$ and Jahn-Teller-undistorted Fe_2O_3, comparing four parameters: bond length distortion index, quadratic elongation, effective coordination number, and van Vleck magnitude $\rho_0 = \sqrt{Q_2^2 + Q_3^2}$. Here, in Figure S3, we show this same data as calculated for CuF_6 octahedra in $KCuF_3$. The behaviour is essentially the same as for NiO_6 octahedra in $NaNiO_2$.

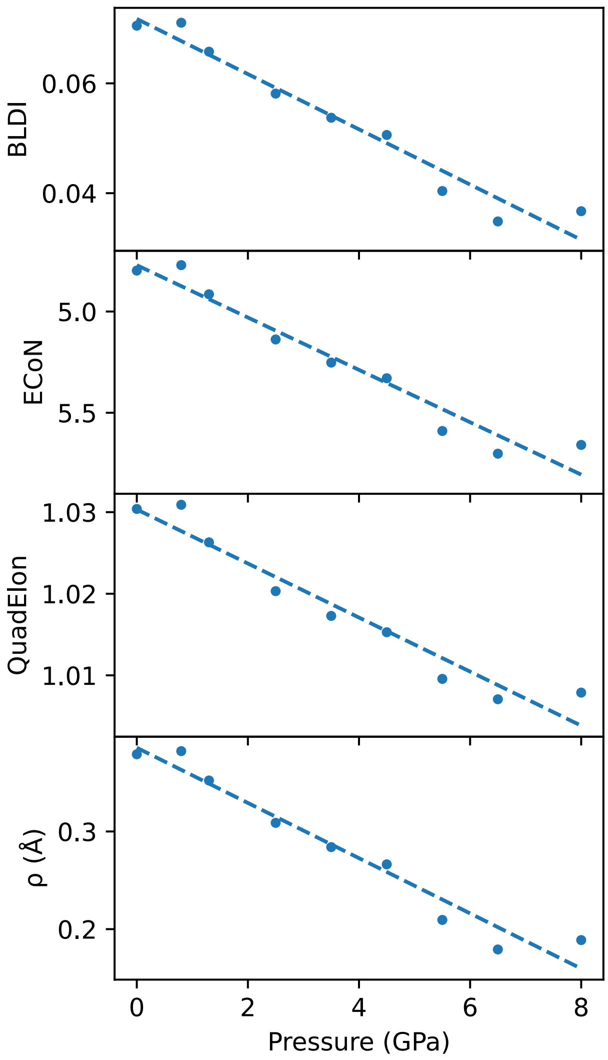

Figure S3: The pressure dependence of bond length distortion index, effective coordination, quadratic elongation, and the van Vleck ρ_0 parameter, for CuF_6 octahedra in $KCuF_3$ as a function of pressure, using crystal structures previously reported in Ref.[12]

Pressure-dependent distortion of NaO_6 octahedra in $NaNiO_2$

In the main text, we showed that the van Vleck parameter $\rho_0 = \sqrt{Q_2^2 + Q_3^3}$ is a more effective parameter for quantifying the magnitude of a Jahn-Teller distortion than similar parameters such as bond length distortion index or effective coordination. While each of these parameters are large for Jahn-Teller-distorted NiO_6 octahedra in $NaNiO_2$ [Figure 7(c)], we show for Fe_2O_3 that, despite a large degree of bond length distortion, ρ_0 remains at zero [Figure 7(d)]. Hence, ρ_0 is only sensitive to distortions with symmetry matching the Jahn-Teller distortion.

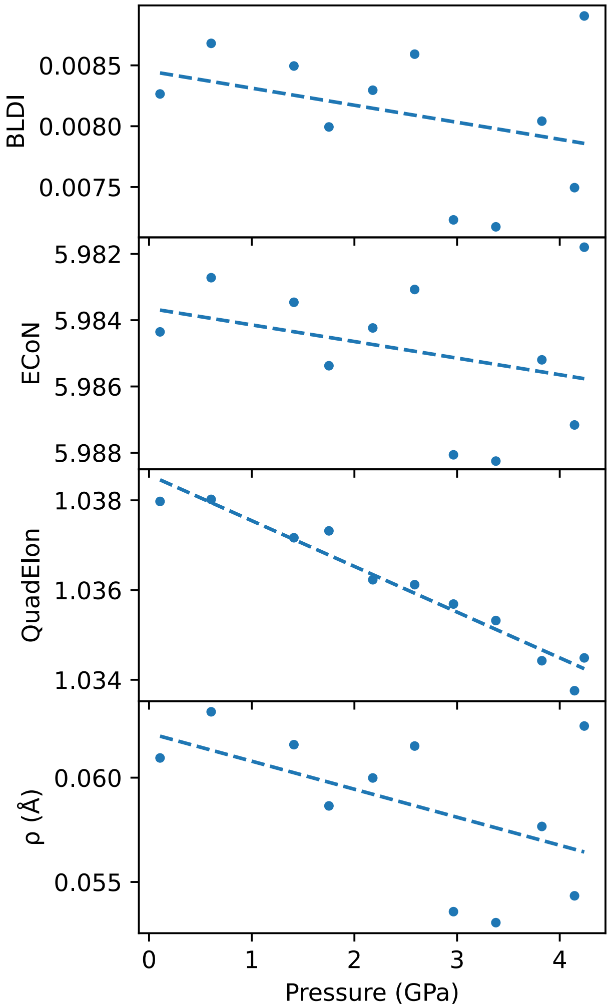

Figure S4: The pressure dependence of bond length distortion index, effective coordination, quadratic elongation, and the van Vleck ρ_0 parameter, for NaO_6 octahedra in $NaNiO_2$ as a function of pressure, using crystal structures previously reported in Ref.[13]

However, we caution that ρ_0 may give a large result for coordination octahedra around Jahn-Teller-inactive cations when the symmetry of the distortion matches the $E_g(Q_2, Q_3)$ Jahn-Teller distortion. Here, we give the example of NaO_6 octahedra in $NaNiO_2$, from the same pressure study referred to in the main text.[13] Figure S4 shows the pressure dependence of the distortion parameters of NaO_6. It can be seen that ρ_0 follows the same trend as the other parameters despite the absence of the Jahn-Teller distortion, due to the NaO_6 octahedra having the same symmetry as its Jahn-Teller-distorted neighbouring NiO_6 octahedra.

Angular shear with pressure and temperature

In the main text, shear distortion is represented in terms of the Q_4 to Q_6 shear modes. The Δ_{shear} modes defined in Equations 14, 16, and 18, also have utility, as these are independent of octahedral size and the level of bond length distortion. For completeness, we plot in Figures S5 and S6 the pressure- and temperature-dependence of these modes, respectively. This shows that angular shear is highly correlated with bond angle standard deviation when shear fraction $\eta \approx 1$ and uncorrelated otherwise.

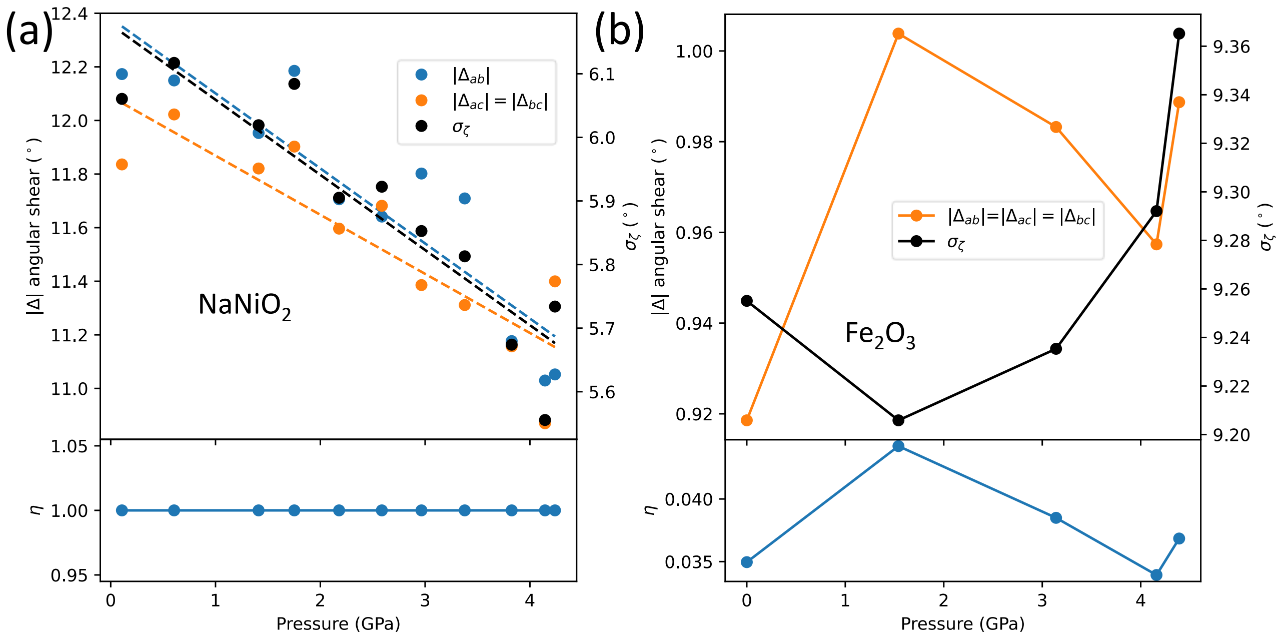

Figure S5: The pressure-dependence of the shear and angular distortion in (a) Jahn-Teller-distorted NiO_6 octahedra in $NaNiO_2$,[13] and (b) Jahn-Teller-undistorted FeO_6 octahedra in Fe_2O_3.[14] Shear distortion is represented with the Δ parameters, and angular distortion is represented by bond angle standard deviation σ_ζ. η is the angular shear fraction. For Fe_2O_3, the average position of the O ligands were taken as the centre of the octahedron. Dashed lines denote linear fits, solid lines connect datapoints.

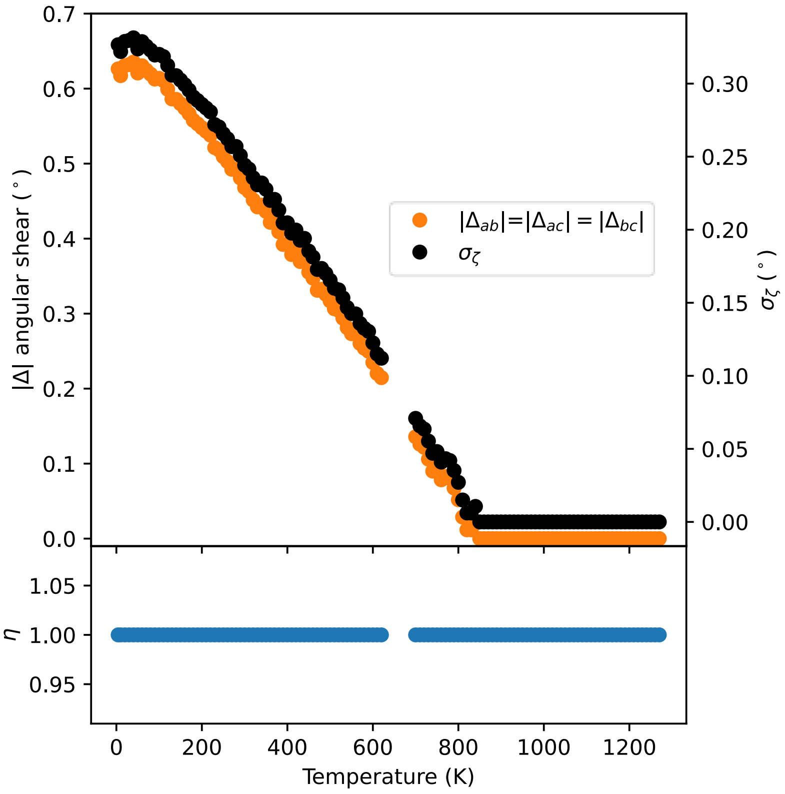

Figure S6: The temperature-dependence of the shear and angular distortion in $LaAlO_3$.[15] Shear distortion is represented with the Δ parameters, and angular distortion is represented by bond angle standard deviation σ_ζ. η is the angular shear fraction.

Graphical explanation of the shear angular modes

Figure S7, below, is a guide to understand the the angles δ_+^a, δ_+^b, δ_-^a, and δ_-^b as described in the main text. These angles are used to calculate Δ_{shear}, $\Delta_{\text{anti-shear}}$, and the shear fraction η. This figure shows the angles for the ab-plane, with similar logic applying for the bc- and ac-planes.

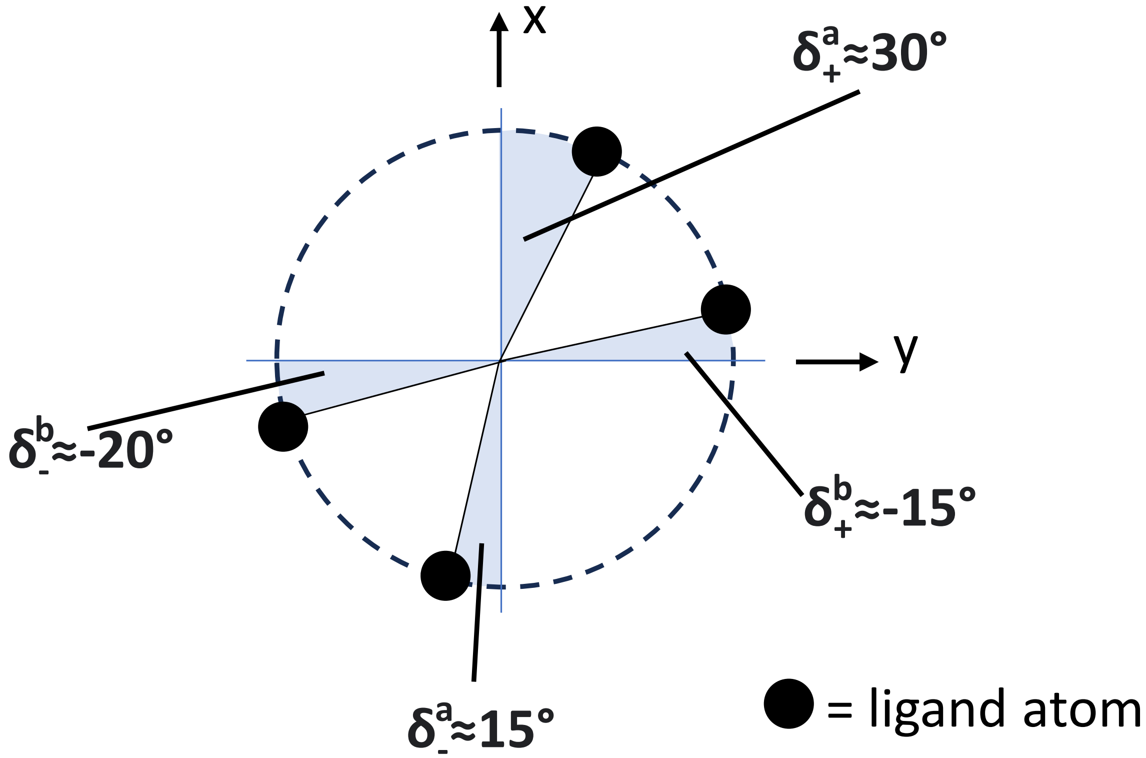

Figure S7: A graphical explanation of the angles δ_+^a, δ_+^b, δ_-^a, and δ_-^b as described in the main text, for an octahedron with the basis shown, within the ab-plane. The origin represents the centre of the octahedron, and the black circles represent ligands.

Tabulated data from the main paper

For reference, we tabulate the data presented in this manuscript. Tables S1, S2, and S3 shows the calculated parameters for CuO_6 octahedra, NiO_6 octahedra, and FeO_6 octahedra in $KCuF_3$, $NaNiO_2$, and Fe_2O_3 respectively, as a function of pressure at room temperature. Table S4 shows a similar table for the variable-temperature structures of $LaAlO_3$ at ambient pressure.

Tables S5 and S6 show the tabulated lattice parameters from Hayward *et al* (2005)[15] used in this study to calculate the octahedral distortion parameters for AlO_6.

P (GPa)	V ($\AA^3$)	D	ECoN	QE	σ^2_ζ ($^{\circ 2}$)	Q_2 ($\AA$)	Q_3 ($\AA$)	Q_4 ($\AA$)	Q_5 ($\AA$)	Q_6 ($\AA$)	ρ_0 ($\AA$)	ϕ ($^\circ$)	Δ_{shear} ($^\circ$)	$\Delta_{\text{antishear}}$ ($^\circ$)	η
∼0	10.818	0.070	4.798	1.030	0	0.358	-0.125	0	0	0	0.379	1.908	0	0	N/A
0.8	10.771	0.071	4.771	1.031	0	0.362	-0.123	0	0	0	0.382	1.897	0	0	N/A
1.3	10.675	0.066	4.915	1.026	0	0.334	-0.113	0	0	0	0.352	1.897	0	0	N/A
2.5	10.599	0.058	5.138	1.020	0	0.291	-0.104	0	0	0	0.309	1.914	0	0	N/A
3.5	10.454	0.054	5.252	1.017	0	0.268	-0.095	0	0	0	0.284	1.912	0	0	N/A
4.5	10.320	0.051	5.329	1.015	0	0.252	-0.088	0	0	0	0.266	1.906	0	0	N/A
5.5	10.259	0.040	5.590	1.010	0	0.193	-0.082	0	0	0	0.209	1.975	0	0	N/A
6.5	10.161	0.035	5.702	1.007	0	0.161	-0.078	0	0	0	0.179	2.022	0	0	N/A
8	9.977	0.037	5.659	1.008	0	0.174	-0.072	0	0	0	0.189	1.962	0	0	N/A

Table S1: Various parameters, calculated for the Cu site at ($\frac{1}{2}$,0,0) using VanVleckCalculator, in $KCuF_3$, as a function of pressure. P is pressure, V is octahedral volume, D is bond length distortion index, ECoN is the effective coordination number, QE is the quadratic elongation, σ^2_ζ is the bond angle variance. For these calculations, the "octahedral_centre" argument was set to "core_atom".

P (GPa)	V ($\mathring{A}^3$)	D	ECoN	QE	σ^2_ζ ($^{\circ 2}$)	Q_2 ($\mathring{A}$)	Q_3 ($\mathring{A}$)	Q_4 ($\mathring{A}$)	Q_5 ($\mathring{A}$)	Q_6 ($\mathring{A}$)	ρ_0 ($\mathring{A}$)	ϕ ($^\circ$)	Δ_{shear} ($^\circ$)	$\Delta_{\text{antishear}}$ ($^\circ$)	η
0.107	10.172	0.051	5.386	1.028	36.730	0	0.265	0.203	0.209	0.209	0.265	0	0.361	0	1
0.605	10.130	0.050	5.411	1.027	37.419	0	0.258	0.203	0.212	0.212	0.258	0	0.365	0	1
1.411	10.104	0.049	5.427	1.026	36.230	0	0.254	0.199	0.208	0.208	0.254	0	0.359	0	1
1.752	10.058	0.050	5.420	1.027	37.016	0	0.256	0.203	0.210	0.210	0.256	0	0.363	0	1
2.179	10.085	0.049	5.441	1.025	34.870	0	0.251	0.195	0.204	0.204	0.251	0	0.352	0	1
2.587	10.056	0.048	5.458	1.025	35.075	0	0.246	0.194	0.205	0.205	0.246	0	0.353	0	1
2.965	10.042	0.049	5.429	1.026	34.254	0	0.253	0.196	0.200	0.200	0.253	0	0.348	0	1
3.378	10.029	0.049	5.437	1.025	33.790	0	0.251	0.195	0.199	0.199	0.251	0	0.346	0	1
3.826	10.050	0.047	5.472	1.024	32.194	0	0.243	0.186	0.196	0.196	0.243	0	0.337	0	1
4.144	10.060	0.048	5.466	1.023	30.864	0	0.245	0.184	0.191	0.191	0.245	0	0.330	0	1
4.239	10.020	0.046	5.502	1.023	32.878	0	0.235	0.184	0.200	0.200	0.235	0	0.341	0	1

Table S2: A table of various parameters, calculated for the Ni site at (0,0,0) using VanVleckCalculator, in $NaNiO_2$, as a function of pressure. P is pressure, V is octahedral volume, D is bond length distortion index, ECoN is the effective coordination number, QE is the quadratic elongation, σ^2_ζ is the bond angle variance. For these calculations, the "octahedral_centre" argument was set to "average_ligand_position".

P (GPa)	V ($\AA^3$)	D	ECoN	QE	σ_ζ^2 ($^{\circ 2}$)	Q_2 ($\AA$)	Q_3 ($\AA$)	Q_4 ($\AA$)	Q_5 ($\AA$)	Q_6 ($\AA$)	ρ_0 ($\AA$)	ϕ ($^\circ$)	Δ_{shear} ($^\circ$)	$\Delta_{\text{antishear}}$ ($^\circ$)	η
~0	10.799	0.042	5.577	1.022	85.657	0	0	-0.020	0.020	-0.020	0	4.583	0.066	0.621	0.011
1.54	10.747	0.043	5.543	1.022	84.748	0	0	-0.021	0.021	-0.021	0	4.590	0.070	0.618	0.013
3.14	10.645	0.040	5.610	1.022	85.291	0	0	-0.021	0.021	-0.021	0	4.591	0.067	0.619	0.012
4.16	10.580	0.038	5.652	1.022	86.342	0	0	-0.021	0.021	-0.021	0	4.591	0.066	0.623	0.011
4.39	10.577	0.039	5.620	1.023	87.707	0	0	-0.021	0.021	-0.021	0	4.590	0.068	0.628	0.012
5.24	10.638	0.048	5.411	1.021	78.195	0	0	-0.028	0.028	-0.028	0	4.629	0.085	0.592	0.020

Table S3: A table of various parameters, calculated for the Fe site at (0,0,~0.144) using VanVleckCalculator, in Fe_2O_3, as a function of pressure. P is pressure, V is octahedral volume, D is bond length distortion index, ECoN is the effective coordination number, QE is the quadratic elongation, σ_ζ^2 is the bond angle variance. For these calculations, the "octahedral_centre" argument was set to "average_ligand_position".

Table S4: A table of various parameters, calculated for the Al site at (0,0,0) using VanVleckCalculator, in $LaAlO_3$, as a function of pressure. P is pressure, V is octahedral volume, D is bond length distortion index, ECoN is the effective coordination number, QE is the quadratic elongation, σ_ζ^2 is the bond angle variance. For these calculations, the "octahedral_centre" argument was set to "average_ligand_position".

T (K)	V ($Å^3$)	D	ECoN	QE	σ_ζ^2 ($°^2$)	Q_2 (Å)	Q_3 (Å)	Q_4 (Å)	Q_5 (Å)	Q_6 (Å)	ρ_0 (Å)	Δ_{shear} (°)	$\Delta_{antishear}$ (°)	η
4.2	9.133	0	6	1	0.107	0	0	-0.010	0.010	-0.010	0	0.019	0	1
10	9.132	0	6	1	0.104	0	0	-0.010	0.010	-0.010	0	0.019	0	1
20	9.135	0	6	1	0.108	0	0	-0.010	0.010	-0.010	0	0.019	0	1
30	9.136	0	6	1	0.109	0	0	-0.010	0.010	-0.010	0	0.019	0	1
40	9.136	0	6	1	0.110	0	0	-0.011	0.011	-0.011	0	0.019	0	1
50	9.133	0	6	1	0.105	0	0	-0.010	0.010	-0.010	0	0.019	0	1
60	9.136	0	6	1	0.108	0	0	-0.010	0.010	-0.010	0	0.019	0	1
70	9.135	0	6	1	0.106	0	0	-0.010	0.010	-0.010	0	0.019	0	1
80	9.135	0	6	1	0.104	0	0	-0.010	0.010	-0.010	0	0.019	0	1
90	9.134	0	6	1	0.102	0	0	-0.010	0.010	-0.010	0	0.019	0	1
100	9.137	0	6	1	0.102	0	0	-0.010	0.010	-0.010	0	0.019	0	1
110	9.136	0	6	1	0.101	0	0	-0.010	0.010	-0.010	0	0.018	0	1
120	9.136	0	6	1	0.098	0	0	-0.010	0.010	-0.010	0	0.018	0	1

Continued on next page

Table S4 – continued from previous page

T (K)	V ($\AA^3$)	D	ECoN	QE	σ_ζ^2 ($^{\circ 2}$)	Q_2 ($\AA$)	Q_3 ($\AA$)	Q_4 ($\AA$)	Q_5 ($\AA$)	Q_6 ($\AA$)	ρ_0 ($\AA$)	Δ_{shear} ($^\circ$)	$\Delta_{\text{antishear}}$ ($^\circ$)	η
130	9.135	0	6	1	0.093	0	0	-0.010	0.010	-0.010	0	0.018	0	1
140	9.137	0	6	1	0.093	0	0	-0.010	0.010	-0.010	0	0.018	0	1
150	9.138	0	6	1	0.091	0	0	-0.010	0.010	-0.010	0	0.018	0	1
160	9.139	0	6	1	0.090	0	0	-0.010	0.010	-0.010	0	0.017	0	1
170	9.138	0	6	1	0.087	0	0	-0.009	0.009	-0.009	0	0.017	0	1
180	9.139	0	6	1	0.085	0	0	-0.009	0.009	-0.009	0	0.017	0	1
190	9.140	0	6	1	0.083	0	0	-0.009	0.009	-0.009	0	0.017	0	1
200	9.141	0	6	1	0.082	0	0	-0.009	0.009	-0.009	0	0.017	0	1
210	9.143	0	6	1	0.080	0	0	-0.009	0.009	-0.009	0	0.016	0	1
220	9.141	0	6	1	0.079	0	0	-0.009	0.009	-0.009	0	0.016	0	1
230	9.143	0	6	1	0.074	0	0	-0.009	0.009	-0.009	0	0.016	0	1
240	9.144	0	6	1	0.073	0	0	-0.009	0.009	-0.009	0	0.016	0	1
250	9.146	0	6	1	0.071	0	0	-0.008	0.008	-0.008	0	0.015	0	1
260	9.146	0	6	1	0.069	0	0	-0.008	0.008	-0.008	0	0.015	0	1
270	9.147	0	6	1	0.066	0	0	-0.008	0.008	-0.008	0	0.015	0	1

Continued on next page

Table S4 – continued from previous page

T (K)	V ($\AA^3$)	D	ECoN	QE	σ_ζ^2 ($^{\circ 2}$)	Q_2 ($\AA$)	Q_3 ($\AA$)	Q_4 ($\AA$)	Q_5 ($\AA$)	Q_6 ($\AA$)	ρ_0 ($\AA$)	Δ_{shear} ($^\circ$)	$\Delta_{\text{antishear}}$ ($^\circ$)	η
280	9.152	0	6	1	0.066	0	0	-0.008	0.008	-0.008	0	0.015	0	1
290	9.151	0	6	1	0.063	0	0	-0.008	0.008	-0.008	0	0.015	0	1
300	9.151	0	6	1	0.060	0	0	-0.008	0.008	-0.008	0	0.014	0	1
310	9.153	0	6	1	0.058	0	0	-0.008	0.008	-0.008	0	0.014	0	1
320	9.154	0	6	1	0.055	0	0	-0.007	0.007	-0.007	0	0.014	0	1
330	9.154	0	6	1	0.053	0	0	-0.007	0.007	-0.007	0	0.013	0	1
340	9.157	0	6	1	0.054	0	0	-0.007	0.007	-0.007	0	0.013	0	1
350	9.159	0	6	1	0.052	0	0	-0.007	0.007	-0.007	0	0.013	0	1
360	9.160	0	6	1	0.048	0	0	-0.007	0.007	-0.007	0	0.013	0	1
370	9.163	0	6	1	0.049	0	0	-0.007	0.007	-0.007	0	0.013	0	1
380	9.163	0	6	1	0.046	0	0	-0.007	0.007	-0.007	0	0.012	0	1
390	9.162	0	6	1	0.042	0	0	-0.007	0.007	-0.007	0	0.012	0	1
400	9.166	0	6	1	0.042	0	0	-0.007	0.007	-0.007	0	0.012	0	1
410	9.166	0	6	1	0.039	0	0	-0.006	0.006	-0.006	0	0.011	0	1
420	9.171	0	6	1	0.040	0	0	-0.006	0.006	-0.006	0	0.012	0	1

Continued on next page

Table S4 – continued from previous page

T (K)	V ($\AA^3$)	D	ECoN	QE	σ_ζ^2 ($^{\circ 2}$)	Q_2 ($\AA$)	Q_3 ($\AA$)	Q_4 ($\AA$)	Q_5 ($\AA$)	Q_6 ($\AA$)	ρ_0 ($\AA$)	Δ_{shear} ($^\circ$)	$\Delta_{\text{antishear}}$ ($^\circ$)	η
430	9.171	0	6	1	0.037	0	0	-0.006	0.006	-0.006	0	0.011	0	1
440	9.174	0	6	1	0.038	0	0	-0.006	0.006	-0.006	0	0.011	0	1
450	9.174	0	6	1	0.034	0	0	-0.006	0.006	-0.006	0	0.011	0	1
460	9.175	0	6	1	0.033	0	0	-0.006	0.006	-0.006	0	0.010	0	1
470	9.175	0	6	1	0.030	0	0	-0.005	0.005	-0.005	0	0.010	0	1
480	9.178	0	6	1	0.030	0	0	-0.006	0.006	-0.006	0	0.010	0	1
490	9.180	0	6	1	0.029	0	0	-0.005	0.005	-0.005	0	0.010	0	1
500	9.181	0	6	1	0.027	0	0	-0.005	0.005	-0.005	0	0.010	0	1
510	9.183	0	6	1	0.026	0	0	-0.005	0.005	-0.005	0	0.009	0	1
520	9.184	0	6	1	0.025	0	0	-0.005	0.005	-0.005	0	0.009	0	1
530	9.186	0	6	1	0.024	0	0	-0.005	0.005	-0.005	0	0.009	0	1
540	9.186	0	6	1	0.022	0	0	-0.005	0.005	-0.005	0	0.009	0	1
550	9.188	0	6	1	0.020	0	0	-0.005	0.005	-0.005	0	0.008	0	1
560	9.188	0	6	1	0.020	0	0	-0.005	0.005	-0.005	0	0.008	0	1
570	9.192	0	6	1	0.018	0	0	-0.004	0.004	-0.004	0	0.008	0	1

Continued on next page

Table S4 – continued from previous page

T (K)	V ($\AA^3$)	D	ECoN	QE	σ^2_ζ ($^{\circ 2}$)	Q_2 ($\AA$)	Q_3 ($\AA$)	Q_4 ($\AA$)	Q_5 ($\AA$)	Q_6 ($\AA$)	ρ_0 ($\AA$)	Δ_{shear} ($^\circ$)	$\Delta_{\text{antishear}}$ ($^\circ$)	η
580	9.194	0	6	1	0.018	0	0	-0.004	0.004	-0.004	0	0.008	0	1
590	9.193	0	6	1	0.017	0	0	-0.004	0.004	-0.004	0	0.008	0	1
600	9.196	0	6	1	0.015	0	0	-0.004	0.004	-0.004	0	0.007	0	1
610	9.197	0	6	1	0.013	0	0	-0.004	0.004	-0.004	0	0.007	0	1
620	9.199	0	6	1	0.013	0	0	-0.004	0.004	-0.004	0	0.006	0	1
700	9.211	0	6	1	0.005	0	0	-0.002	0.002	-0.002	0	0.004	0	1
710	9.213	0	6	1	0.004	0	0	-0.002	0.002	-0.002	0	0.004	0	1
720	9.215	0	6	1	0.004	0	0	-0.002	0.002	-0.002	0	0.004	0	1
730	9.215	0	6	1	0.003	0	0	-0.002	0.002	-0.002	0	0.003	0	1
740	9.215	0	6	1	0.002	0	0	-0.001	0.001	-0.001	0	0.003	0	1
750	9.216	0	6	1	0.002	0	0	-0.002	0.002	-0.002	0	0.003	0	1
760	9.217	0	6	1	0.002	0	0	-0.001	0.001	-0.001	0	0.002	0	1
770	9.219	0	6	1	0.002	0	0	-0.001	0.001	-0.001	0	0.003	0	1
780	9.221	0	6	1	0.002	0	0	-0.001	0.001	-0.001	0	0.002	0	1
790	9.221	0	6	1	0.001	0	0	-0.001	0.001	-0.001	0	0.002	0	1

Continued on next page

Table S4 – continued from previous page

T (K)	V ($\mathring{A}^3$)	D	ECoN	QE	σ^2_ζ ($^{\circ 2}$)	Q_2 ($\mathring{A}$)	Q_3 ($\mathring{A}$)	Q_4 ($\mathring{A}$)	Q_5 ($\mathring{A}$)	Q_6 ($\mathring{A}$)	ρ_0 ($\mathring{A}$)	Δ_{shear} ($^\circ$)	$\Delta_{\text{antishear}}$ ($^\circ$)	η
800	9.221	0	6	1	0.001	0	0	-0.001	0.001	-0.001	0	0.002	0	1
810	9.222	0	6	1	0	0	0	0	0	0	0	0.001	0	1
820	9.221	0	6	1	0	0	0	0	0	0	0	0	0	1
830	9.221	0	6	1	0	0	0	0	0	0	0	0	0	1
840	9.226	0	6	1	0	0	0	0	0	0	0	0.001	0	1
850	9.227	0	6	1	0	0	0	0	0	0	0	0	0	N/A
860	9.227	0	6	1	0	0	0	0	0	0	0	0	0	N/A
870	9.233	0	6	1	0	0	0	0	0	0	0	0	0	N/A
880	9.237	0	6	1	0	0	0	0	0	0	0	0	0	N/A
890	9.240	0	6	1	0	0	0	0	0	0	0	0	0	N/A
900	9.244	0	6	1	0	0	0	0	0	0	0	0	0	N/A
910	9.247	0	6	1	0	0	0	0	0	0	0	0	0	N/A
920	9.250	0	6	1	0	0	0	0	0	0	0	0	0	N/A
930	9.254	0	6	1	0	0	0	0	0	0	0	0	0	N/A
940	9.257	0	6	1	0	0	0	0	0	0	0	0	0	N/A

Continued on next page

Table S4 – continued from previous page

T (K)	V ($\mathring{A}^3$)	D	ECoN	QE	σ_ζ^2 ($^{\circ 2}$)	Q_2 ($\mathring{A}$)	Q_3 ($\mathring{A}$)	Q_4 ($\mathring{A}$)	Q_5 ($\mathring{A}$)	Q_6 ($\mathring{A}$)	ρ_0 ($\mathring{A}$)	Δ_{shear} ($^\circ$)	$\Delta_{\text{antishear}}$ ($^\circ$)	η
950	9.260	0	6	1	0	0	0	0	0	0	0	0	0	N/A
960	9.264	0	6	1	0	0	0	0	0	0	0	0	0	N/A
970	9.267	0	6	1	0	0	0	0	0	0	0	0	0	N/A
980	9.270	0	6	1	0	0	0	0	0	0	0	0	0	N/A
990	9.274	0	6	1	0	0	0	0	0	0	0	0	0	N/A
1000	9.277	0	6	1	0	0	0	0	0	0	0	0	0	N/A
1010	9.280	0	6	1	0	0	0	0	0	0	0	0	0	N/A
1020	9.284	0	6	1	0	0	0	0	0	0	0	0	0	N/A
1030	9.286	0	6	1	0	0	0	0	0	0	0	0	0	N/A
1040	9.290	0	6	1	0	0	0	0	0	0	0	0	0	N/A
1050	9.294	0	6	1	0	0	0	0	0	0	0	0	0	N/A
1060	9.297	0	6	1	0	0	0	0	0	0	0	0	0	N/A
1070	9.300	0	6	1	0	0	0	0	0	0	0	0	0	N/A
1080	9.303	0	6	1	0	0	0	0	0	0	0	0	0	N/A
1090	9.307	0	6	1	0	0	0	0	0	0	0	0	0	N/A

Continued on next page

Table S4 – continued from previous page

T (K)	V ($\AA^3$)	D	ECoN	QE	σ_ζ^2 ($^{\circ 2}$)	Q_2 ($\AA$)	Q_3 ($\AA$)	Q_4 ($\AA$)	Q_5 ($\AA$)	Q_6 ($\AA$)	ρ_0 ($\AA$)	Δ_{shear} ($^\circ$)	$\Delta_{\text{antishear}}$ ($^\circ$)	η
1100	9.310	0	6	1	0	0	0	0	0	0	0	0	0	N/A
1110	9.313	0	6	1	0	0	0	0	0	0	0	0	0	N/A
1120	9.317	0	6	1	0	0	0	0	0	0	0	0	0	N/A
1130	9.321	0	6	1	0	0	0	0	0	0	0	0	0	N/A
1140	9.324	0	6	1	0	0	0	0	0	0	0	0	0	N/A
1150	9.327	0	6	1	0	0	0	0	0	0	0	0	0	N/A
1160	9.331	0	6	1	0	0	0	0	0	0	0	0	0	N/A
1170	9.334	0	6	1	0	0	0	0	0	0	0	0	0	N/A
1180	9.337	0	6	1	0	0	0	0	0	0	0	0	0	N/A
1190	9.341	0	6	1	0	0	0	0	0	0	0	0	0	N/A
1200	9.344	0	6	1	0	0	0	0	0	0	0	0	0	N/A
1210	9.348	0	6	1	0	0	0	0	0	0	0	0	0	N/A
1220	9.351	0	6	1	0	0	0	0	0	0	0	0	0	N/A
1230	9.355	0	6	1	0	0	0	0	0	0	0	0	0	N/A
1240	9.358	0	6	1	0	0	0	0	0	0	0	0	0	N/A

Continued on next page

Table S4 – continued from previous page

T (K)	V ($\AA^3$)	D	ECoN	QE	σ_ζ^2 ($^{\circ 2}$)	Q_2 ($\AA$)	Q_3 ($\AA$)	Q_4 ($\AA$)	Q_5 ($\AA$)	Q_6 ($\AA$)	ρ_0 ($\AA$)	Δ_{shear} ($^\circ$)	$\Delta_{\text{antishear}}$ ($^\circ$)	η
1250	9.361	0	6	1	0	0	0	0	0	0	0	0	0	N/A
1260	9.365	0	6	1	0	0	0	0	0	0	0	0	0	N/A
1270	9.368	0	6	1	0	0	0	0	0	0	0	0	0	N/A

Table S5: A table of the lattice parameters and O positions for rhombohedral $LaAlO_3$ with space group $R\bar{3}c$. For the unlisted lattice parameters: $b = a$, $\alpha = \beta = 90°$, and $\gamma = 120°$. The La sites are at $(0, 0, \frac{1}{4})$, the Al sites are at $(0, 0, 0)$, and the O sites are at $(O_z, 0, \frac{1}{4})$. These results are taken from Hayward *et al* (2005).[15]

T (K)	a (Å)	c (Å)	O(z)
4.2	5.35977	13.086	0.5288
10	5.35981	13.0863	0.5285
20	5.35975	13.0861	0.529
30	5.35978	13.0864	0.5291
40	5.35975	13.0862	0.5292
50	5.35977	13.0865	0.5287
60	5.35979	13.0867	0.5291
70	5.35991	13.0871	0.5289
80	5.35989	13.0875	0.5288
90	5.36	13.088	0.5286
100	5.36029	13.089	0.5287
110	5.36027	13.089	0.5286
120	5.36047	13.0906	0.5284
130	5.36057	13.0913	0.528
140	5.36084	13.0925	0.5281
150	5.36098	13.0934	0.528
160	5.36118	13.0945	0.5279
170	5.36136	13.0953	0.5277
180	5.36153	13.0964	0.5275
190	5.36195	13.0978	0.5274
200	5.36213	13.0987	0.5273

Continued on next page

Table S5 – continued from previous page

T (K)	a (Å)	c (Å)	O(z)
210	5.36233	13.1	0.5273
220	5.36232	13.1	0.5271
230	5.36286	13.1025	0.5267
240	5.36313	13.1037	0.5267
250	5.36366	13.1053	0.5264
260	5.36382	13.1064	0.5263
270	5.36425	13.108	0.526
280	5.36475	13.11	0.5262
290	5.36511	13.1113	0.5258
300	5.3654	13.1126	0.5254
310	5.36574	13.1139	0.5253
320	5.36619	13.1158	0.525
330	5.36655	13.1171	0.5247
340	5.36689	13.1183	0.5249
350	5.3674	13.1202	0.5247
360	5.36793	13.1221	0.5242
370	5.36837	13.1237	0.5244
380	5.36881	13.1253	0.5239
390	5.3691	13.1267	0.5233
400	5.36959	13.1286	0.5235
410	5.37003	13.1302	0.523
420	5.37034	13.1317	0.5234
430	5.37087	13.1335	0.5229
440	5.37124	13.1347	0.5231

Continued on next page

Table S5 – continued from previous page

T (K)	a (Å)	c (Å)	O(z)
450	5.37176	13.1367	0.5225
460	5.37216	13.138	0.5222
470	5.37256	13.1397	0.5216
480	5.37291	13.141	0.5218
490	5.37331	13.1428	0.5217
500	5.37376	13.1444	0.5214
510	5.37438	13.1464	0.521
520	5.37456	13.1472	0.521
530	5.37521	13.1493	0.5206
540	5.37563	13.1509	0.5201
550	5.37602	13.1526	0.5199
560	5.37603	13.1527	0.5199
570	5.37696	13.1559	0.5195
580	5.37737	13.1577	0.5194
590	5.37737	13.1578	0.5192
600	5.37827	13.1608	0.5186
610	5.37865	13.1628	0.5181
620	5.37915	13.1647	0.518
700	5.38233	13.1795	0.5155
710	5.38265	13.1815	0.5153
720	5.38312	13.1829	0.5151
730	5.38342	13.1845	0.5143
740	5.38391	13.1867	0.5135
750	5.38462	13.187	0.513

Continued on next page

Table S5 – continued from previous page

T (K)	a (Å)	c (Å)	O(z)
760	5.38499	13.1889	0.5123
770	5.38586	13.1893	0.5118
780	5.38641	13.1907	0.5116
790	5.38703	13.1919	0.5101
800	5.38756	13.1938	0.5087
810	5.38761	13.197	0.508
820	5.38808	13.1985	0.5056
830	5.38808	13.1985	0.5056
840	5.38997	13.1997	0.5026

Table S6: A table of the lattice parameters and O positions for cubic $LaAlO_3$ with space group $Pm\bar{3}m$. For the angular lattice parameters, $\alpha = \beta = \gamma = 90°$. The La sites are at $(\frac{1}{2}, \frac{1}{2}, \frac{1}{2})$, the Al sites are at $(0, 0, 0)$, and the O sites are at $(\frac{1}{2}, 0, 0)$. These results are taken from Hayward *et al* (2005).[15]

T (K)	$a = b = c$ (Å)
850	3.8113
860	3.81131
870	3.81216
880	3.81269
890	3.81307
900	3.8136
910	3.814
920	3.81447
930	3.81495
940	3.81534
950	3.81581
960	3.81631
970	3.81675
980	3.81714
990	3.81767
1000	3.81811
1010	3.81854
1020	3.81905
1030	3.81944
1040	3.81992
1050	3.82042

Continued on next page

Table S6 – continued from previous page

T (K)	$a = b = c$ (Å)
1060	3.82085
1070	3.82132
1080	3.82176
1090	3.82223
1100	3.82268
1110	3.82314
1120	3.82366
1130	3.82413
1140	3.82457
1150	3.82501
1160	3.82553
1170	3.82597
1180	3.82636
1190	3.8269
1200	3.8273
1210	3.82782
1220	3.82833
1230	3.82878
1240	3.82923
1250	3.82968
1260	3.83019
1270	3.83062

References

(1) Harris, C. R. et al. Array programming with NumPy. *Nature* **2020**, *585*, 357–362, DOI: 10.1038/s41586-020-2649-2.

(2) Ong, S. P.; Richards, W. D.; Jain, A.; Hautier, G.; Kocher, M.; Cholia, S.; Gunter, D.; Chevrier, V. L.; Persson, K. A.; Ceder, G. Python Materials Genomics (pymatgen): A robust, open-source python library for materials analysis. *Computational Materials Science* **2013**, *68*, 314–319, DOI: 10.1016/j.commatsci.2012.10.028.

(3) Hunter, J. D. Matplotlib: A 2D graphics environment. *Computing in Science & Engineering* **2007**, *9*, 90–95, DOI: 10.1109/MCSE.2007.55.

(4) Swanson, D. K.; Peterson, R. C. Polyhedral volume calculations. *The Canadian Mineralogist* **1980**, *18*, 153–156.

(5) Baur, W. H. The geometry of polyhedral distortions. Predictive relationships for the phosphate group. *Acta Crystallographica Section B: Structural Crystallography and Crystal Chemistry* **1974**, *30*, 1195–1215.

(6) Robinson, K.; Gibbs, G.; Ribbe, P. Quadratic elongation: a quantitative measure of distortion in coordination polyhedra. *Science* **1971**, *172*, 567–570.

(7) Hoppe, R. Effective coordination numbers (ECoN) and mean fictive ionic radii (MEFIR). *Zeitschrift für Kristallographie-Crystalline Materials* **1979**, *150*, 23–52.

(8) Koçer, C. P.; Griffith, K. J.; Grey, C. P.; Morris, A. J. Cation disorder and lithium insertion mechanism of Wadsley–Roth crystallographic shear phases from first principles. *Journal of the American Chemical Society* **2019**, *141*, 15121–15134.

(9) Halasyamani, P. S. Asymmetric cation coordination in oxide materials: Influence of lone-pair cations on the intra-octahedral distortion in d^0 transition metals. *Chemistry of Materials* **2004**, *16*, 3586–3592.

(10) Kanamori, J. Crystal distortion in magnetic compounds. *Journal of Applied Physics* **1960**, *31*, S14–S23.

(11) Thygesen, P. M. M.; Young, C. A.; Beake, E. O. R.; Romero, F. D.; Connor, L. D.; Proffen, T. E.; Phillips, A. E.; Tucker, M. G.; Hayward, M. A.; Keen, D. A., et al. Local structure study of the orbital order/disorder transition in $LaMnO_3$. *Physical Review B* **2017**, *95*, 174107.

(12) Zhou, J.-S.; Alonso, J. A.; Han, J. T.; Fernández-Díaz, M.; Cheng, J.-G.; Goodenough, J. B. Jahn-Teller distortion in perovskite $KCuF_3$ under high pressure. *Journal of Fluorine Chemistry* **2011**, *132*, 1117–1121.

(13) Nagle-Cocco, L. A. V.; Bull, C. L.; Ridley, C. J.; Dutton, S. E. Pressure Tuning the Jahn–Teller Transition Temperature in $NaNiO_2$. *Inorganic Chemistry* **2022**, *61*, 4312–4321.

(14) Finger, L. W.; Hazen, R. M. Crystal structure and isothermal compression of Fe_2O_3, Cr_2O_3, and V_2O_3 to 50 kbars. *Journal of Applied Physics* **1980**, *51*, 5362–5367.

(15) Hayward, S.; Morrison, F. D.; Redfern, S.; Salje, E.; Scott, J.; Knight, K.; Tarantino, S.; Glazer, A.; Shuvaeva, V.; Daniel, P., et al. Transformation processes in $LaAlO_3$: Neutron diffraction, dielectric, thermal, optical, and Raman studies. *Physical Review B* **2005**, *72*, 054110.
